# Genome reconstruction of the non-culturable spinach downy mildew *Peronospora effusa* by metagenome filtering

**DOI:** 10.1371/journal.pone.0225808

**Published:** 2020-05-12

**Authors:** Joël Klein, Manon Neilen, Marcel van Verk, Bas E. Dutilh, Guido Van den Ackerveken

**Affiliations:** 1 Department of Biology, Plant-Microbe Interactions, Utrecht University, Utrecht, The Netherlands; 2 Crop Data Science, KeyGene, Wageningen, The Netherlands; 3 Department of Biology, Theoretical Biology and Bioinformatics, Utrecht University, Utrecht, The Netherlands; Tianjin University, CHINA

## Abstract

*Peronospora effusa* (previously known as *P*. *farinosa f*. *sp*. *spinaciae*, and here referred to as *Pfs*) is an obligate biotrophic oomycete that causes downy mildew on spinach (*Spinacia oleracea*). To combat this destructive many disease resistant cultivars have been bred and used. However, new *Pfs* races rapidly break the employed resistance genes. To get insight into the gene repertoire of *Pfs* and identify infection-related genes, the genome of the first reference race, *Pfs1*, was sequenced, assembled, and annotated. Due to the obligate biotrophic nature of this pathogen, material for DNA isolation can only be collected from infected spinach leaves that, however, also contain many other microorganisms. The obtained sequences can, therefore, be considered a metagenome. To filter and obtain *Pfs* sequences we utilized the CAT tool to taxonomically annotate ORFs residing on long sequences of a genome pre-assembly. This study is the first to show that CAT filtering performs well on eukaryotic contigs. Based on the taxonomy, determined on multiple ORFs, contaminating long sequences and corresponding reads were removed from the metagenome. Filtered reads were re-assembled to provide a clean and improved *Pfs* genome sequence of 32.4 Mbp consisting of 8,635 scaffolds. Transcript sequencing of a range of infection time points aided the prediction of a total of 13,277 gene models, including 99 RxLR(-like) effector, and 14 putative Crinkler genes. Comparative analysis identified common features in the predicted secretomes of different obligate biotrophic oomycetes, regardless of their phylogenetic distance. Their secretomes are generally smaller, compared to hemi-biotrophic and necrotrophic oomycete species. We observe a reduction in proteins involved in cell wall degradation, in Nep1-like proteins (NLPs), proteins with PAN/apple domains, and host translocated effectors. The genome of *Pfs1* will be instrumental in studying downy mildew virulence and for understanding the molecular adaptations by which new isolates break spinach resistance.

## Introduction

Phytopathogenic oomycetes are eukaryotic microbes that infect a large range of plant species. Due to their hyphal infection structures they appear fungal-like, however, taxonomically they belong to the Stramenopiles [1]. The most devastating phytopathogenic oomycetes are found within the orders of *Albuginales*, *Peronosporales* and *Pythiales*.

The highly radiated *Peronosporales* order contains species with different lifestyles. The most infamous species of this order are in the hemi-biotrophic *Phytophthora* genus. Other species within the *Peronosporales* are the obligate biotrophic downy mildews that cause disease while keeping the plant alive. The relationships between downy mildews and *Phytophthora* species have long been unresolved [2]. Until recently, downy mildew species were underrepresented in studies addressing oomycete phylogeny. This is mainly because the obligate biotrophic nature of the species makes them hard to work with and they are, therefore, under-sampled compared to other oomycete phytopathogens.

The first phylogenetic trees based on morphological traits and single gene comparisons [3, 4] classified the downy mildews as a sister clade to the *Phytophthora* species within the order of Peronosporales. Recently published studies using multiple gene and full genome comparisons, including a number of downy mildew species, suggest that the downy mildews have multiple independent origins within the *Phytophthora* genus [2, 5, 6].

The downy mildew *Peronospora effusa* (previously known as *P*. *farinosa forma specialis spinaciae*, and here referred to as *Pfs*), is the most important pathogen of spinach. *Pfs* affects the leaves, severely damaging the harvestable parts of the spinach crop. Under favorable environmental conditions, *Pfs* infection can progress rapidly resulting in abundant sporulation within a week post inoculation that is visible as a thick grey ‘furry layer’ of sporangiophores producing abundant asexual spores [7] Preventing spread of this pathogen is difficult, since only a few fungicides are effective in chemical control [8]. As a result, the disease can cause severe losses in this popular crop, and infected fields often completely lose their market value.

During infection, hyphae of *Pfs* grow intercellularly through the tissue and locally breach through cell walls to allow the formation of haustoria [9]. These invaginating feeding structures form a platform for the intimate interaction between plant and pathogen cells, and function as a site for the exchange of nutrients, signals and proteins. Oomycetes deliver proteins into plant cells to alter host immunity [10], thereby escaping and suppressing plant immune responses [11]. These and other molecules are secreted by pathogens to promote the establishment and maintenance of a successful infection in the host are called effectors. Effector proteins can either be functional outside the plant cells (apoplastic effectors) or inside plant cells (host-translocated effectors). Two types of host translocated are known in oomycetes; the RXLR and crinkler (CRN) effectors. They are characterized by the presence of a signal peptide, a conserved domain at the N-terminus and a variable C-terminal part which is responsible for the function of the effector in the cell [12–14].

Here we describe the sequencing of genomic DNA obtained from *Pfs* spores collected from infected spinach plants using a combination of Illumina and PacBio sequencing. Sequencing of obligate biotrophic species is complicated as the spore washes of infected plant leaves contain many other microorganisms. Bioinformatic filtering on taxonomy using the recently developed Contig Annotation Tool CAT [15] was deployed to remove the majority of contaminating sequences. The obtained assembly of race *Pfs1* was used to predict genes and compare its proteome, in particular its secretome, with that of other oomycete taxa. We show that the secretomes of obligate biotrophic oomycetes are functionally more similar to each other than to that of more closely related species with a different lifestyle.

## Materials and method

### Downy mildew infection

*Peronospora effusa* race 1 (*Pfs1*) was provided by the Dutch breeding company Rijk Zwaan Breeding BV in 2014. As *Pfs1* is an obligate biotrophic maintenance was done on *Spinacia oleracea* Viroflay plants. Seeds were sown on soil, stratified for two days at 4°C and grown under long day condition for two weeks (16h light, 70% humidity, 21°C). sporangiophores were washed off infected plant material in 50 ml Falcon tubes. The solution was filtered through miracloth and the spore concentration was checked under the microscope. Four-day-old *Spinacia oleracea* Viroflay plants were infected with *Pfs* by spraying a spore solution (70 spores/ul) in tap water. Seven days post inoculation, *Pfs* sporangiospores were collected from heavily-infected spinach leaves with tap water, using a soft brush to prevent plant and soil contamination and used for DNA isolation and genome sequencing.

### DNA isolation and genome sequencing

The sporangiospores were freeze-dried, ground and dissolved in CTAB (Cetyltrimethyl ammonium bromide) extraction buffer, lysed for 30 minutes at 65°C, followed by a phenol-chloroform/isoamyl-alcohol, and chloroform/isoamyl-alcohol extraction. DNA was precipitated from the aqueous phase with NaOAc and ice-cold isopropanol. The precipitate was collected by centrifugation, and the resulting pellet washed with ice cold 70% ethanol. DNA was further purified using a QIAGEN Genomic-tip 20/G, following the standard protocol provided by the manufacturer. DNA was quantified using a Qubit HS dsDNA assay (Thermo Fisher Scientific) and sheared using the Covaris S220 ultrasonicator set to 550 bp. The sequencing library was constructed with the Illumina TruSeq DNA PCR-Free kit. Fragment size distribution in the library was determined before and after the library preparation using the Agilent Bioanalyzer 2100 with HS-DNA chip (Agilent Technologies). The library was sequenced on an Illumina Nextseq machine in high output mode with a 550 bp genomic insert paired end 150 bp reads. Illumina reads with low quality ends were trimmed (Q<36) using prinseq-lite [16].

For PacBio sequencing the input DNA was amplified by WGA (Whole Genome Amplification) using the Illustra GenomiPhi V2 DNA Amplification (GE Healthcare). The sequencing library for PacBio was constructed according to the manufacturer protocol. The resulting library was sequenced on 24 SMRT cells (P6 polymerase and C4 chemistry) using the RSII sequencer (KeyGene N.V., Wageningen). The obtained PacBio reads were error-corrected using the FALCON pipeline [17] with the standard settings using the SMRT Portal that is part of the SMRT analysis software package version 2.3.0 from PacBio [18]. The analysis software package was installed according to the installation instructions on an Amazon WebService (AWS) cloud-based computer and operated via its build in GUI.

### Taxonomic classification of long reads

The taxonomic origin of each error corrected PacBio read was determined using the CAT (Contig Annotation Tool) pipeline version 1.0 with default parameters [15]. To do this, CAT first identifies open reading frames (ORFs) on the long sequences or contigs using Prodigal [19] and queries them against the NCBI non-redundant (nr) protein database (retrieved November 2016) using DIAMOND [20]. A benchmarked weighting scheme is then applied that allows the contig to be classified with high precision [15].

### Genome assembly and identification of repeats

A pre-assembly was made using taxonomically filtered and corrected PacBio sequences and 60% of the Illumina reads using SPAdes version 3.5.0 [21]. The error-corrected PacBio reads were used as long reads in the assembly, SPAdes was set to use *k*-mer lengths of: 21, 33, 55, 77, 99, 127 for the assembly and the—careful option was used to minimize the number of mismatches in the final contigs. The contigs derived from the pre-assembly were filtered using the CAT tool (see above), and sequences that were designated as bacterial or non-stramenopile eukaryotes were collected. The entire set of Illumina sequencing reads were aligned to the collection of removed sequences (annotated as bacterial and non-stramenopile) with Bowtie version 2.2.7 using default settings [22]. Illumina reads that aligned to these sequences were removed from the Illumina data set. The remaining Illumina reads (Illumina filtered), and PacBio sequences were re-assembled with SPAdes (same settings as the preassembly), which resulted in a final *Pfs1* genome assembly. A custom repeat library for the *Pfs1* genome assembly was generated with RepeatModeler [23]. Repeat regions in the assembled *Pfs1* genome were predicted using RepeatMasker 4.0.7 [23].

### Quality evaluation of the assembly

*K-m*ers of length 21 in the filtered Illumina data set were counted with Jellyfish count version 2.0 [24] with settings -C -m 21 -s 1000000000 followed by Jellyfish histo. The histogram was plotted with GenomeScope [25] to produce a graphical output and an estimate of the genome size. The coverage of the genome by PacBio sequences was determined by aligning the unfiltered error-corrected PacBio reads to the *Pfs1* genome assembly using BWA-mem [26] and selected–x pacbio option. The BBmap pileup [27] script was used to determine the percentage covered bases by PacBio reads in the final assembly of the *Pfs1*.

The GC-content per contig larger than 1kb was calculated using a Perl script [28]. GC density plots were generated in Rstudio version 1.0.143 using GGplot version 3.1 [29]. For comparison, the same analysis was done on a selection of other publicly available oomycete assemblies; *Hyaloperonospora (H*.*) arabidopsidis* [30], *Peronospora (P*.*) belbahrii* [31], *Phytophthora (Ph*.*) infestans* [32], *Bremia (B*.*) lactucae* [33], *Phytophthora parasitica [34]*, *Phytophthora ramorum* (Pr102) [34], *Phytophthora sojae* [34], *Peronospora tabacina* (968-S26) [35] and *Plasmopara (Pl*.*) viticola* [5].

Kaiju [36] was used to analyze the taxonomic origin by mapping reads to the NCBI nr nucleotide database (November 2017). The input for Kaiju was generated using ART [37] set at 20x coverage with 150 bp Illumina to create artificial sequencing reads from the various FASTA assembly files of the genomes of different oomycetes.

Genome completeness and gene duplications were analyzed with BUSCO version 3 [38] with default settings using the protists Ensembl database (May 2018).

#### RNA sequencing and gene model prediction

RNA of *Pfs1* at different stages during the infection was isolated and sequenced to aid gene model prediction. Infected leaves and cotyledons were harvested every day from three days post infection (dpi) until sporulation (7 dpi). Besides these infected leaves, spores were harvested, and a subset of these spores were placed in a petri dish with water and incubated overnight at 16° C to allow them to germinate. RNA was isolated using the RNeasy Plant Mini Kit from Qiagen, and the RNA was analyzed using the Agilent 2100 bioanalyzer to determine the RNA quality and integrity. The RNA-sequencing libraries were made with the Illumina TruSeq Stranded mRNA LT kit. Paired-end 150 bp reads were obtained from the different samples with the Illumina Nextseq 500 machine on high output mode. RNA-seq reads from all the samples were pooled, aligned to the *Pfs1* assembly using Tophat [39], and used as input for gene model prediction using BRAKER1 [40]. The obtained gene models for the *Pfs1* genome together with the RNA-seq alignment result, the repeat models, and results obtained from a BLASTp search to the nr NCBI database (January 2017), were loaded into a locally-installed WebApollo [41] instance. Gene models on the 100 largest contigs of the genome were manually curated and all gene models were exported from WebApollo for further use.

#### Gene annotation and the identification of functional domains

Bedtools intersect version 2.27 was used to determine the overlap between *Pfs1* gene models and annotated repeat elements in the genome. Gene models that had more than 20% overlap with a region marked as a repeat-containing gene. ANNIE [42] was used to annotate proteins on the *Pfs1* genome based on Pfam domains [43] and homologous sequences in the NCBI-Swissprot database (accessed Augustus 2017). Sequences that were annotated as transposon*s* by ANNIE were removed from the gene set. SignalP 4.1 [44] was used to predict the presence and location of a signal peptide, the D-cutoff for noTM and TM networks were set at 0.34 to increase sensitivity [45]. TMHMM version 2 [46] was used to predict the presence of transmembrane helices in the proteins of *Pfs1*. To identify proteins that possess one or more WY domains an HMM model made by Win *et al*. [47] was used. Protein sequences that possessed a WY domain were extracted and realigned. This alignment was used to construct a new HMM model using HMMER version 3.2.1 [48] and queried again against all protein models in the *Pfs* genome to obtain the full set of WY domains containing proteins.

#### Effector identification

Putative effectors residing on the genome of *Pfs1* were identified with a custom- made pipeline [49] constructed using the Perl [50] scripting language. Secreted proteins were screened for the occurrence of known translocation domains within the first 100 amino acids after the signal peptide. Proteins with a canonical RxLR, or a degenerative RxLR (xxLR or RxLx) combined with either an EER-like or a WY domain or both where considered putative RxLR effectors. A degenerative EER domain was allowed to vary from the canonical EER by at most one position.

Proteins with a canonical LFLAK motif or a degenerative LFLAK and HVL motif in the first 100 amino acids of the protein sequence. A HMMer profile was constructed based on the LFLAK or HVL containing proteins. This HMMer profile was used to identify Crinkler effector candidates lacking the LFLAK or HVL motif.

Proteins with an additional transmembrane domain or a C-terminal ER retention signal (H/KDEL) were removed. WY domains were identified using hmmsearch version 3.1b2 [51] with the published *Phytophthora* HMM model (see above) [52]. *Pfs* WY-motif containing protein sequences were realigned and used to construct a *Pfs* specific WY HMM model using hmmbuild version 3.1b2 [51]. Based on the *Pfs* specific HMM model WY-motif containing *Pfs* proteins were determined.

The effector prediction for the comparative analysis was done in a similar fashion, except the published *Phytophthora* HMM model for RxLR prediction and a published model for CRN prediction was used [53]. The prediction of effectors using the same model in each species enabled the comparison.

#### Comparative gene distance analysis

Based on the gene locations encoded in the GFF file the 3’ and 5’ intergenic distances between genes on contigs were calculated as a measure of local gene density. When a gene is located next to beginning or end of a contig, the distance was taken from the start or end of the gene to the end of the contig. Putative high confidence RxLR effector sequences that encode for proteins with either an exact canonical RxLR motif or an RxLR-like motif in combination with one or more WY-motifs were selected for the comparison (66 in total). Distances were visualized using a heat map constructed with the GGPlot geom_hex function [29]. Statistical significance was determined using the Wilcoxon signed-rank test [54].

#### Comparative secretomics

The predicted proteomes of eighteen plant pathogenic oomycetes were obtained from Ensembl and NCBI (S1 Table). Proteins in the collected proteomes that have a predicted secretion signal [44] (SignalP v.4.1, D-cutoff for SignalP-noTM and TM networks = 0.34 [45]), no additional transmembrane domain (TMHMM 2.0 [46]) or C-terminal K/HDEL domain were considered secreted. Functional annotations of the secreted proteins were predicted using InterProScan [55] and the CAZymes database [56] using the dbCAN2 meta server [57].

#### Phylogenetic analysis

The phylogenetic relationships between the proteomes of the studied species were inferred using Orthofinder [58]. Orthofinder first identifies ‘orthogroups’ of proteins that descended from a single ancestral protein. Next it determines pairwise orthologs between each pair of species. Orthogroups with only one protein of each species were used to make gene trees using MAFFT [59]. The species tree was inferred from the gene trees using the distance algorithms of FastMe [60] and visualized using EvolView v2 [61].

#### Principal component analysis

The total number of InterPro and CAZymes domain per species was summarized in a counts table. For each domain the number was divided by the total number of domains for that species. The normalized matrix has been loaded into Phyloseq version 1.22.3 [62] with R version 3.4.4 [63] in RStudio [64]. A PCA plot has been made with the Phyloseq ordinate function on euclidean distance. The PCA plot has been made with the GGPlot R package [29]. The biplot has been generated with the standard prcomp function in R with the same normalized matrix. Figures were optimized using Adobe Photoshop 2017.01.1.

#### Permutational analysis of variance (PERMANOVA)

A PERMANOVA using distance matrices was used to statistically test whether there is a difference between the clades based on their CAZymes and InterPro domains. PERMANOVA is a non-parametric method for multivariate analysis of variance using permutations. The data has been double root transformed with the vegdist function from the R-package vegan version 2.5–3 [65]. After the transformation the PERMANOVA has been calculated with the adonis function from the Vegan package. A total number of 999 permutations have been made to retrieve a representative permutation result.

#### Enrichment analysis

A chi-square test with Bonferroni correction was used to identify under- and over-represented Pfam domains in each group (*Hyaloperonospora/Peronospora*, *Plasmopara*, *Albugo*) compared to *Phytophthora*. The actual range was the sum of the proteins that have a given domain. The expected range was the fraction of proteins with a given domain that is expected to belong to a species cluster giving the overall ratio of Pfam domains between species clusters.

## Results

An early race 1 isolate, *Pfs1*, of *Peronospora effusa* was used to create a reference genome as it predates resistance breeding in spinach and its infection is effectively stopped by all spinach resistance genes known to date. Race 1 was first identified in 1824 [66]. Since downy mildews cannot be grown axenically we isolated asexual sporangiospores by carefully washing highly-infected leaves of the universally susceptible cultivar Viroflay. Genomic DNA was isolated from freeze-dried spores and used to construct libraries for PacBio and Illumina sequencing, resulting in 1.09 million PacBio reads with a N50 of 9,253 bp, and 535 million Illumina reads of 150 bp. The paired-end Illumina reads were used for a trial assembly using Velvet. Inspection of the draft assembly showed that many contigs were of bacterial instead of oomycete origin. This is likely caused by contamination of the isolated *Pfs* spores with other microorganisms that reside on infected leaves and that are collected in the wash-offs. We, therefore, decided to treat the sequences as a metagenome and bioinformatically filter the sequences and corresponding reads.

### Taxonomic filtering

**Fig 1:**
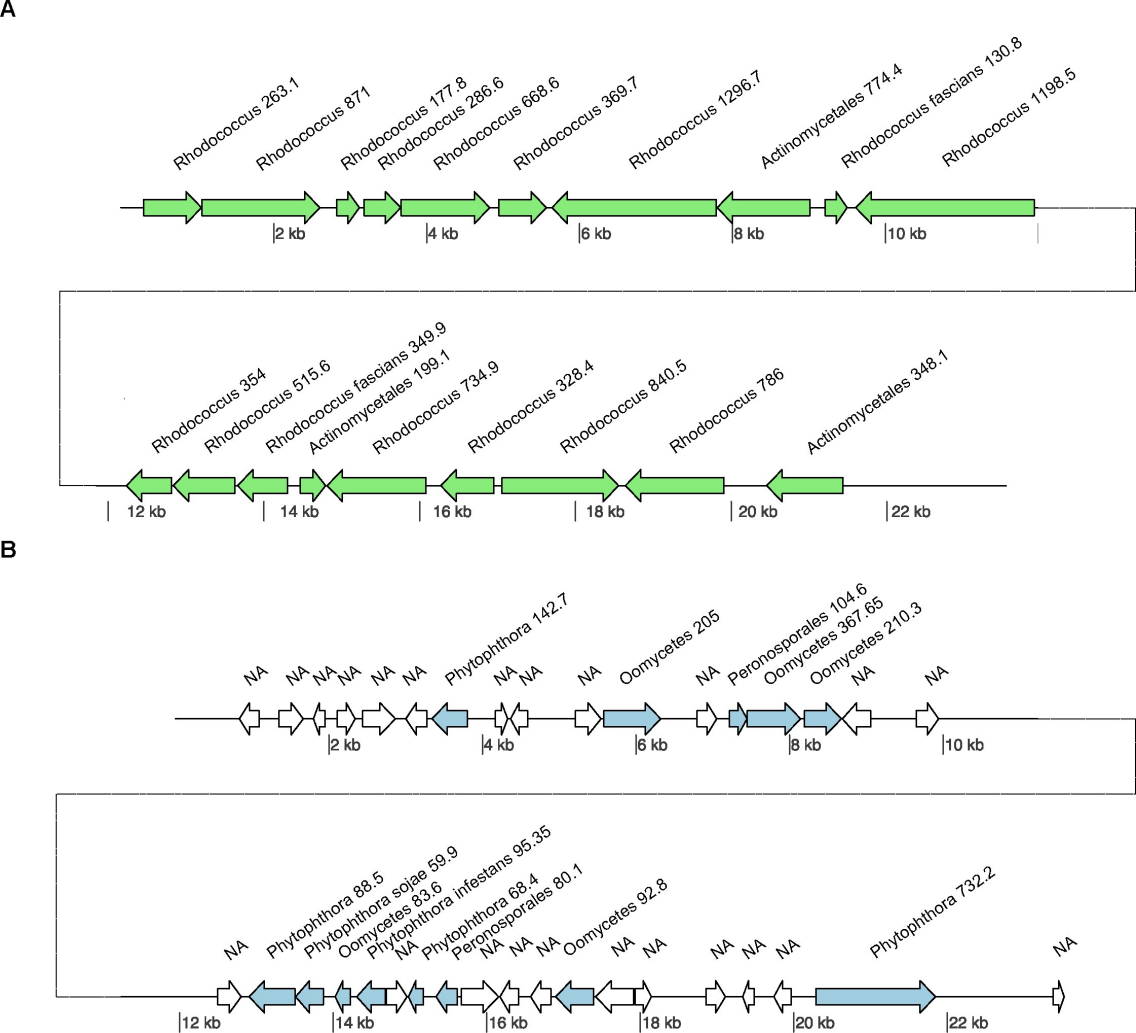
Taxonomic classification by CAT. Two contigs are depicted and per ORF a single top hit is shown. (A) Contig from the pre-assembly assigned by the CAT tool as bacterial, ORFs of bacterial origin are colored green, and ORF with no hits to the database are colored white. On this contig most ORFs had a highest blast hit with *Rhodococcus* species. The ΣBmax for this contig is 10982. and the highest ΣBtaxon is for the *Rhodococcus* genus at 9660, which is well above the cutoff of 5491 (ΣBmax * 0.5). The taxonomic origin of this contig was therefore assigned to the genus *Rhodococcus*, and as a consequence this contig was regarded as non-*Pfs* and removed. (B) Contig from the pre-assembly assigned by the CAT tool as an oomycete contig. On this contig all ORFs have a best hit to an oomycete species, and the ΣBmax is 2328. In fact, most ORFs have a best hit to species in the *Phytophthora* genus (ΣBtaxon: 1184), or the Peronosporales family (ΣBtaxon: 184). The ΣBtaxon for the *Phytophthora* genus is above the cutoff at 1164 (ΣBmax *0.5) thus assigning this contig to the *Phytophthora* genus, and consequently this contig is maintained for the *Pfs* genome assembly.

To filter out the sequences that could be classified as contaminants we deployed CAT [15] on long reads and contigs derived from assemblies. Details on the CAT method are described in the materials and methods section. In short, CAT utilizes the combined taxonomic annotations of multiple individual ORFs found on each sequence to determine its likely taxonomic origin. This allows for a robust taxon classification that is based on multiple hits, rather than a single best hit. An example of the CAT taxonomic classification for two of our sequences (contigs) is visualized in Fig 1.

**Fig 2:**
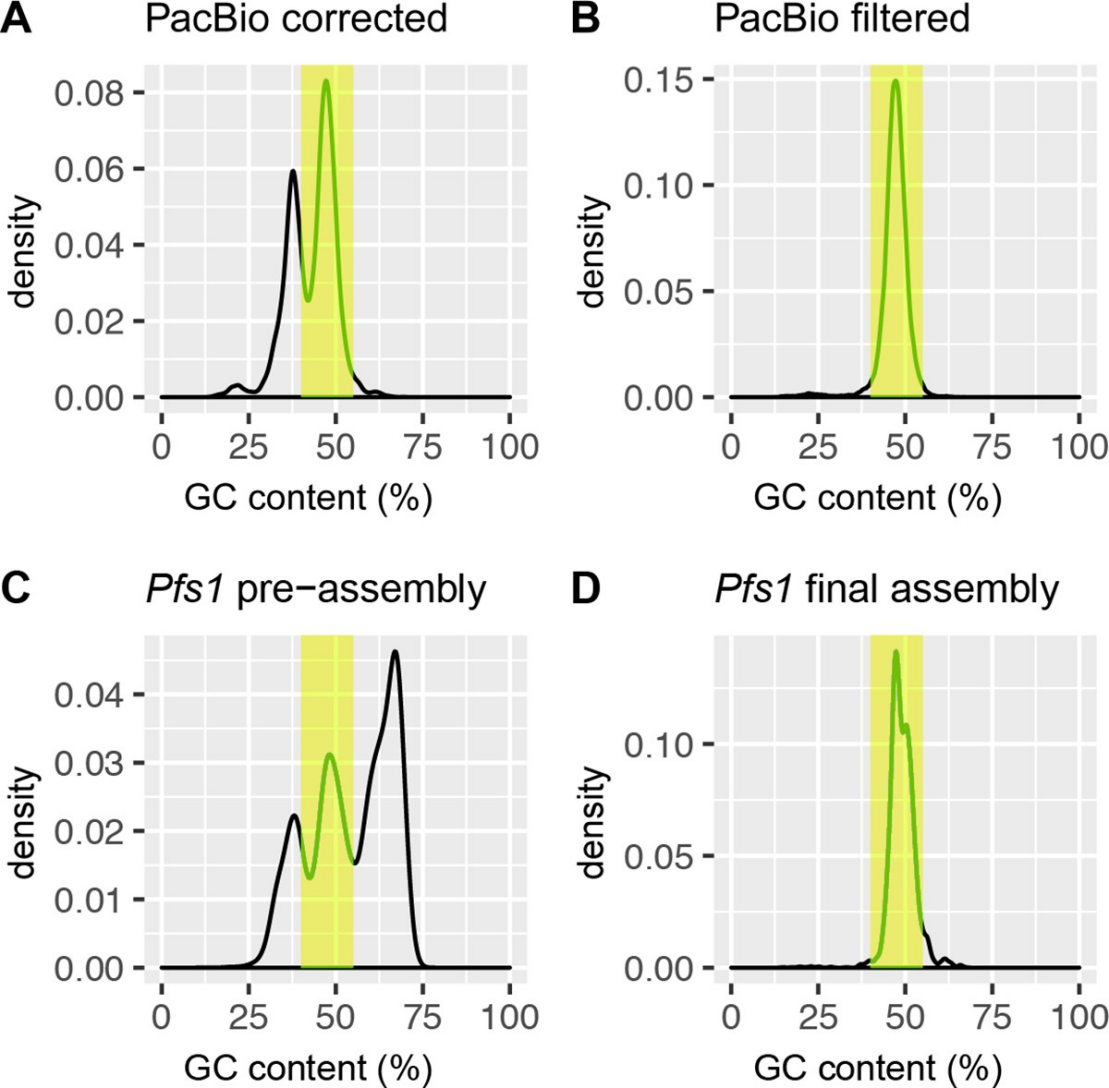
Density plot of the GC values of PacBio reads and assembly before and after CAT filtering of sequences. The yellow bar indicates the region between 40 and 55% GC, based on reads >1 kb. (A) PacBio reads before CAT-filtering show a bimodal distribution with a presumed peak of contaminating sequences with a GC content of ~40%. (B) PacBio reads after CAT-filtering show a distribution consisting of a single peak with a GC content around ~46%. (C**)**. GC content of the *Pfs1* contigs from the pre-assembly before filtering shows additional peaks at around 30 and 60 GC%, indicating that there are many contaminant contigs. (D) GC content of the *Pfs1* contigs after filtering of the reads with the CAT tool shows that the additional peaks are no longer present and have thus been successfully filtered out.

CAT was first used on the long PacBio reads. As these reads contain about 15% base call errors on average, they were first error-corrected using the FALCON pipeline. The FALCON pipeline fixes long PacBio reads by mapping short reads obtained in the same runs. The resulting 466,225 PacBio reads had a total length of 1,003 Mb with a N50 of 3,325 bp and were subsequently assigned a taxonomic classification using CAT. PacBio reads that were classified as prokaryotic, or non-stramenopile eukaryotic (e.g. Fungi) were removed, whereas reads with the assigned taxonomy “stramenopiles” or “unknown” were retained. This resulted in a cleaned set of 232,846 PacBio reads with a total length of 522 Mb with a N50 of 3,458 bp that was used for a hybrid pre-assembly. In order to evaluate the effectiveness of the CAT tool in removing contaminating genomic sequences we analyzed the GC-content of the reads. The corrected PacBio reads showed two distinct peaks (Fig 2A), whereas oomycete genomes have a GC band-width around 50%, as shown in S1A Fig for the contigs of the *Phytophthora infestans* genome [32]. After CAT filtering a single peak remained with a narrow GC-content distribution around ~48%, demonstrating that the tool, that does not take into account GC-content but uses a weighting scheme based on protein sequence similarity, was effective in removing contaminating sequences (Fig 2B).

### Hybrid assembly

A hybrid pre-assembly was generated using the genome assembler SPAdes that can combine long PacBio with short Illumina reads. The input consisted of all corrected and filtered PacBio reads together with 60% randomly extracted Illumina reads (321 Million read, 96.3 Gb, to decrease assembly run time and memory requirements). The pre-assembly consisted of 170,143 contigs with a total length of 176 Mb and an N50 of 6,446 bp, of which only 21,690 contigs were larger than 1 kb. CAT filtering was applied to the contigs of the pre-assembly, CAT marked 16,518 contigs consisting of 91.5 Mb (52% of total assembled bases) as contaminant sequences. Next, Illumina reads were aligned to these and Illumina read-pairs of which at least one end aligned were removed from the data set. A final assembly was generated with the CAT-filtered PacBio and remaining 77.6 million Illumina reads, resulting in 8,635 scaffolds with a total length of 32.4 Mb. The assembly size corresponds with the estimate genome size of 36,2 Mb that was determined based on *k*-mer count frequency (Table 1) in the filtered Illumina reads.

**Table 1:** Summary of statistics for the hybrid assembly of the *Pfs1* genome.

	*Pfs1* final	*Pfs1* size-filtered
Assembly size	32.40 Mb	30.48 Mb
GC content	47.75%	47.80%
Longest scaffold	310.10 kb	310.10 kb
Repeat size	6.93 Mb	6.38 Mb
# Contigs	8,635	3,608
N50	32,837 bp	36,273 bp
# Gene models	13,227	12,630
	***k*-mer estimation**	
Assembly size	36.18 Mb	
Repeat size	8.76 Mb	
Read Error Rate	1.04%	

Data is provided for the final assembly (*Pfs1* final) and size-filtered assembly omitting the contigs smaller than 1 kb (*Pfs1* filtered). In addition, genome information based on *k*-mer counting of the Illumina reads is provided, giving an estimate for the predicted genome size and repeat content.

### Filtering results

The effect of filtering with CAT on the pre-assembly is well visualized by plotting the GC-content of the contigs (Fig 2C), similar as for the PacBio reads. In the pre-assembly many contigs with a GC-percentage deviating from the 40–55% range are present, indicating that it contains many contaminating sequences. After filtering, the final assembly shows one major peak of the expected GC-content at ~48%, with a minor shoulder of slightly higher GC-content (Fig 2D).

**Fig 3:**
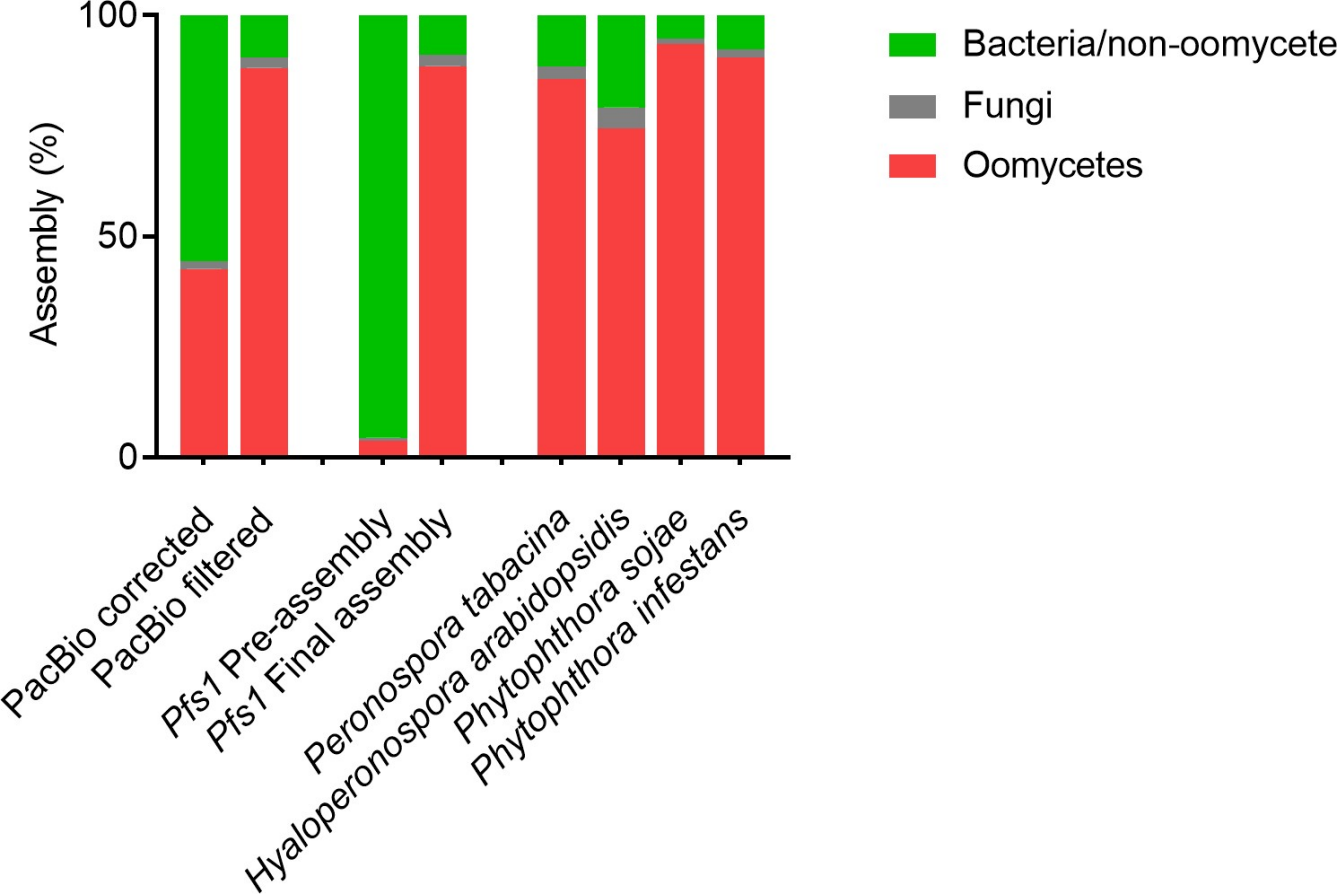
Taxonomic classification of reads in assemblies of different oomycetes. Kaiju bar plot showing the percentage of reads assigned to three taxonomical classes; Oomycetes, Fungi and Bacteria and other non-oomycetes. In error corrected PacBio reads 42.64% are assigned to oomycetes, after filtering with CAT 88.09% of the reads are assigned to oomycetes. For the pre-assembly (96.3 Gb), only 5% of the artificial reads is assigned to oomycetes. For the *Pfs1* final assembly (32.4 Mb), 88.6% of the reads are assigned to oomycetes. This is comparable to other oomycetes that can be axenically grown on plates, indicating that the remaining non-oomycete-assigned sequences are most likely a result of an incorrect classification in the database.

To assess the effectiveness of the taxonomic filtering we used Kaiju [36] as a complementary tool. Kaiju is typically used for taxonomic classification of sequencing reads in metagenome analysis but here we used it to determine the effect of taxonomic filtering by CAT. For this, genome assemblies of *Pfs1* and other oomycetes were divided into artificial short reads. The taxonomic distributions generated by Kaiju provide a clear picture of the removal of contaminating sequences from the *Pfs1* genome data (Fig 3). Whereas the pre-assembly mostly contained artificial reads with an assigned bacterial taxonomy, this was reduced to 14% in the final assembly. The percentage of >80% of oomycete-assigned reads in the *Pfs1* final assembly is similar to what we observe for the high-quality genome assemblies of *P*. *infestans* and *P*. *sojae*, pathogens that can be grown axenically, i.e. free of contaminating other microbes (Fig 3).

### Genome statistics

To assess the quality of the assembly we re-aligned the Illumina reads to the contigs and found a large variation in coverage between the contigs smaller than 1 kb and the larger contigs, suggesting that these small contigs contain a high number of repeats or assembly errors. In addition, the CAT pipeline depends on classification of individual ORFs on contigs, so it’s accuracy may be expected to improve with contig length. Therefore, several small contigs could possibly be derived from microbes other than *Pfs*. Removing contigs smaller than 1 kb (5027 contigs) resulted in a small reduction of 1.9 Mb in genome length, slightly reducing the assembly size to 30.5 Mb, but resulting in a 58% reduction in the number of contigs. The remaining 3608 contigs, larger than 1 kb, had an N50 of 36,273 bp. The statistics of the size-filtered assembly are further detailed in Table 1.

To assess the gene space completeness of our assembly in comparison to other oomycete genomes we used BUSCO that identifies single core orthologs that are conserved in a certain lineage. Here, we used the protist Ensembl database as the protist lineage encompasses the oomycetes and other Stramenopila. According to the BUSCO analysis the gene space in our final assembly is 88.9% complete with only 0.5% fragmented genes and 0.5% duplicates. This gene space completeness score is similar to that of other downy mildew genomes, but slightly lower than of genomes of *Phytophthora* species (S2 Table). Furthermore, the low number of duplicates suggests that there is a low incidence of erroneous assembly of haplotypes, suggesting that the obtained *Pfs* assembly represents most of the single-copy gene space of the *Pfs* genome [38].

### Repeat content

In addition to a genome size estimate, the *k*-mer analysis estimated a repeat content of ~8.8 Mb. This is slightly higher than the observed repeat content in the final assembly of ~6.9 Mb (~6.4 Mb in the size-filtered assembly) (Table 1). The difference between the estimated repeat size and the repeat content in the assembly (1.87 Mb) is most likely caused by long repetitive elements that are hard to assemble. Repeatmasker [23] identified a total of 13,089 repeat elements of which most are part of the Gypsy and Copia superfamily. We also identified 562 LINEs (Long interspersed nuclear elements) and only 16 SINE (short interspersed nuclear elements), which belong to the class I transposon (retrotransposons). Other repeat elements consisted of 2297 simple repeats, 298 Low complexity regions, 391 different types of DNA transposons (Table 2), and several (278) other minor repeat types; full details can be found in S3 Table.

**Table 2:** Total number and size of major repeat types identified in the *Pfs1* genome assembly.

Repeat type	Count	% of total count	Total length (bp)
LTR	9247	70,65	6532069
LINE	562	4,29	201127
Simple repeat	2297	17,55	97983
DNA repeats/TE	391	2,99	46677
Rolling Circle TE	97	0,74	26123

The percentage of total count is based on the total number of repeat types identified in the assembly which can be found in S3 Table.

When we compare the genome assembly size of *Pfs* (30.5 Mb) to other sequenced oomycete genomes such as those of *Ph*. *infestans* (240 Mb), *H*. *arabidopsidis* (100 Mb), *Pl*. *halstedii* (75.3 Mb) or the relatively small genome of *P*. *tabacina* (63.1 Mb), *Pfs* has a strikingly compact genome (S4 Table). The repeat content (21%) is also low compared to that of other oomycetes, e.g. *Ph*. *infestans* (74%), *H*. *arabidopsidis* (43%), *Pl*. *halstedii* (39% Mbp) and more comparable to *P*. *tabacina* (24%).

### *Pfs* gene prediction

#### RNA sequencing

Gene prediction is greatly aided by transcript sequence information. We, therefore, isolated and sequenced mRNA from *Pfs* spores and *Pfs*-infected spinach leaves at several time points during the infection. For this, leaves were harvested daily starting from 3 days post inoculation (dpi) until 7 dpi when sporulation was observed. In addition, mRNA was also isolated from sporangiospores and germlings grown from spores that were incubated in water overnight. The 7 different samples ensure a broad sampling of transcripts to facilitate gene identification. Illumina transcript sequences (659 million) were aligned to the assembled *Pfs* genome which resulted in ~100 million aligned read pairs. Most of the other reads map to the spinach genome but were not further analyzed.

#### Predicted proteins

The aligned transcript read pairs served as input for the BRAKER1 [40] pipeline to generate a *Pfs* specific training set for gene model prediction. This was then used to predict 13227 gene models on the final assembly. The corresponding protein models were annotated using ANNIE [42] and provided putative annotations for 7297 *Pfs* proteins (S5 Table). We found that 12630 protein models reside on contigs larger than 1 kb and are thus contained in the size-filtered assembly. In addition, we found that 2983 gene models had 20% or more overlap with a repeat that was identified by RepeatMasker [23], another 952 protein models were annotated by ANNIE as transposable elements. When analyzing protein models that reside on small contigs (<1 kb) we observe that most of them (61%) have a significant overlap with a repeat region and are marked by ANNIE as transposons. The number of gene models found in the assembly of *Pfs1* is strikingly low in comparison to that in *Ph*. *infestans* (17,792), *H*. *arabidopsidis* (14,321), *Pl*. *halstedii* (15,469) and more similar to *P*. *tabacina* (11,310).

#### Secretome and host-translocated effectors

For the identification of the *Pfs* secretome as well as of candidate host-translocated RxLR and Crinkler effectors we choose to start with the proteins encoded by the initial 13,227 gene set. This reduced the risk of missing effectors that are encoded on smaller contigs (< 1 kb). SignalP [44] prediction identified 783 proteins with a N-terminal signal peptide. Of these, 231 were found to have an additional transmembrane domain (as determined by TMHMM [46] analysis) leaving 557 proteins. In addition, five of these carried a C-terminal H/KDEL motif that functions as an ER retention signal. The resulting set of 552 secreted proteins, ~ 4% of the *Pfs1* proteome, was used for secretome comparison.

Previous research showed that some effectors of the lettuce downy mildew *Bremia lactucae* have a single transmembrane domain in addition to the signal peptide [67]. Therefore, we chose to predict the host-translocated effectors not only from the secretome but also from the set of proteins with a signal peptide and an additional transmembrane domain. A total of 99 putative RxLR or RXLR-like proteins and 14 putative Crinkler effectors were identified (S2 and S3). Ten putative RxLR effector proteins were found to have a single transmembrane domain. Also, five putative RxLR effectors were found on contigs smaller than 1 kb (S6 Table). Of the 99 RxLR effectors, 64 had a canonical RxLR domain, while 35 had a degenerative RxLR domain combined with an EER-like and/or WY domain [68]. The number of host-translocated effectors in *Pfs* is significantly smaller compared to that of *Phytophthora* species (eg. 563 RxLR and 385 effector genes in the genomes of *P*. *infestans* [32] and *P*. *sojae* [69] respectively). Crinkler effectors are charaterized by the N-terminal five amino acid “LFLAK” domain [14]. Five of the identified putative Crinkler effectors had a canonical LFLAK domain. The others had a degenerative LFLAK combined with an HVL domain or were identified using the custom made Crinkler HMM.

#### Genomic distribution of effectors

**Fig 4:**
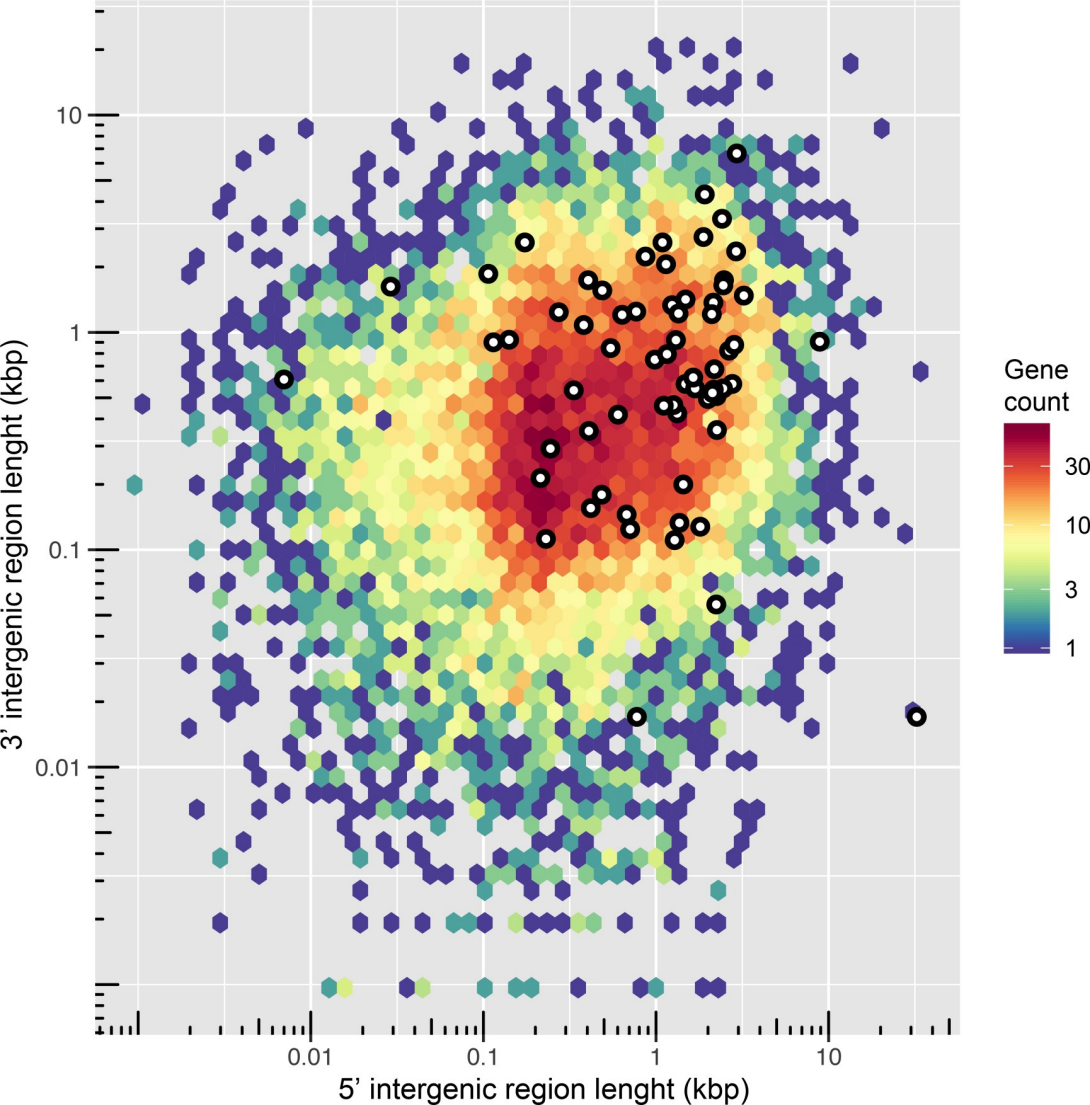
Genome spacing of predicted genes of *Pfs1*. The distance between neighbouring genes was depicted by plotting the 5′ and 3′ intergenic distances (on a log10 scale) for each if the 13,227 predicted genes. The scale bar represents the number of genes in each bin, shown as a color-coded hexagonal heat map in which red indicates a gene dense and blue a gene-poor region. The locations of putative *Pfs* effectors genes are indicated with white dots.

It was previously described for the potato late blight pathogen *P*. *infestans* that effectors often reside in genomic regions with a relatively large repeat content compared the rest of genome [70]. To test this in *Pfs*, the distance between neighboring genes was measured to estimate the genomic context of the 13277 *Pfs1* genes in general and for 66 selected RxLR effector (canonical RxlR and degenerative RxLR with WY-motifs) genes specifically. To get a good overview of the intergenic distances we plotted the 3’ and 5’ values for all the genes in the *Pfs1* genome on a log10 scaled heat map (Fig 4).

The genome of *Pfs1* is highly gene dense and effectors show a modest but significant (Wilcoxon rank sum test, p = 1.914e^-11^) enrichment in the gene-spare regions of the genome (Fig 4). The median 3’ and 5’ combined spacing for all genes is 925 bp, while for the selected effector genes it is 2976 bp. However, the difference in gene density between the effectors and core genes is not as strong as in the *P*. *infestans* two-speed genome [32].

### Comparative analysis of orthologs

**Fig 5:**
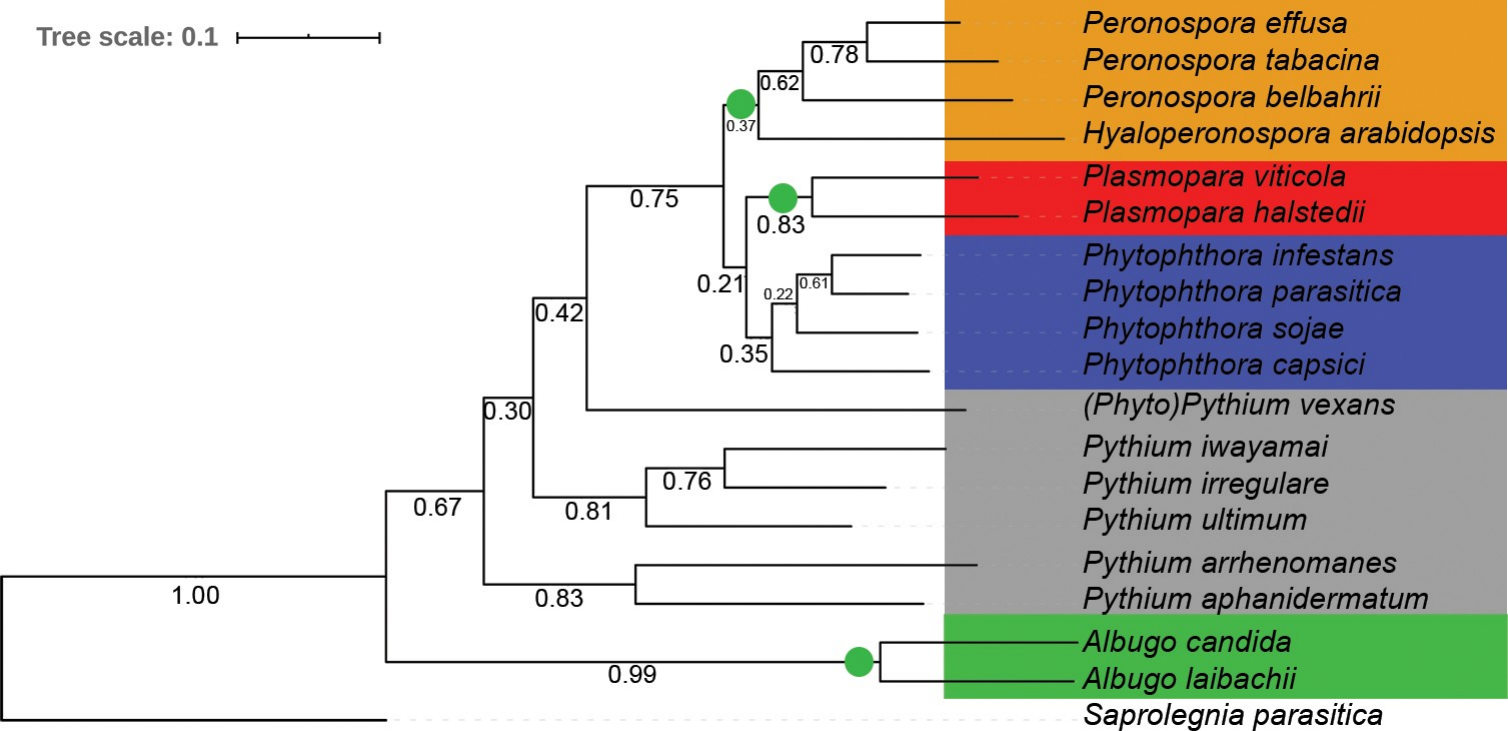
Maximum likelihood tree of 18 plant infecting oomycete species based on core othologous proteins. The tree was inferred from 152 single copy ortholog groups in which all species in the comparison where represented. Branch numbers represent bootstrap values of N = 12171 trees. Five taxonomic clusters were defined for further analysis; *Hyaloperonospora/Peronospora* (green), *Plasmopara* (red), *Phytophthora* (blue), *Pythium* (grey) and *Albugo* (green). The obligate biotrophic clades are highlighted using green circle.The fish infecting species *Saprolegnia parasitica*, was used as an outgroup.

Eighteen phytopathogenic oomycete species, that represent a diverse taxonomic range and different lifestyles, were chosen for a comparative analysis with *Pfs* (Table 3). The objective of the comparison is to see whether the biotrophic lifestyle of downy mildew species, like *Pfs*, is reflected in the secretome. For the analysis, the secretome of *Pfs* was compared to that of closely related *Phytophthora* (hemibiotrophic), *Plasmopara* (biotrophic) and more distantly related *Pythium* (necrotrophic) and *Albugo* (biotrophic) species. First, the predicted proteins of each species were used to create a multigene phylogenetic tree to infer their taxonomic relationships using Orthofinder. In total, 86.9% (267,813) of all proteins were assigned to 14,484 orthogroups. Of those, 2383 had proteins from all species in the dataset of which 152 groups contained proteins corresponding to single copy proteins in each species. These single-copy orthologous proteins of each species were used to infer a Maximum-likelihood species tree (Fig 5).

**Table 3:** Predicted secretomes of 18 oomycete species used in this study.

* *	Predicted proteins	Secretome	% secreted
*P*. *effusa*	13227	552	4,2
*P*. *belbahrii*	9049	494	4,7
*H*. *arabidopsidis*	14321	999	7
*P*. *tabacina*	18447	798	4,3
*Pl*. *halstedii*	15498	1071	6,9
*Pl*. *viticola*	12201	1850	15,2
*Ph*. *infestans*	18138	1885	10,4
*Ph*. *parasitica*	27942	2250	8,1
*Ph*. *sojae*	26584	2337	8,8
*Ph*.*capsici*	19805	1433	7,2
*Py*. *arrhenomanes*	13805	913	6,6
*Py*. *aphanidermatum*	12312	928	7,5
*Py*. *irregulare*	13805	961	7
*Py*. *iawyamai*	15249	1067	7
*Py*. *vexans*	11958	863	7,2
*Py*. *ultimum*	15322	1071	7
*A*. *candida*	13310	888	6,8
*A*. *laibachii*	13804	679	4,9

The total number of predicted proteins, those with a signal peptide (SP), proteins with SP but without additional transmembrane domains (TM), and the number of proteins with SP, no TM, and no C-terminal KDEL sequence are shown. In the final column the percentage of the proteome that is predicted to be secreted is highlighted.

The resulting tree shows that *Pfs* clusters with *H*. *arabidopsidis* (*Hpa*), *P*. *tabacina* (*Pta*) and *P*. *belbahrii* (*Pbe*). The closest relative of *Pfs*, in this study, based on single-copy orthologs is the downy mildew of tobacco *Pta*, followed by the basil-infecting *Pbe*. Based on the tree, *Hpa* is more divergent from the former three downy mildew species within the *Hyaloperonospora/Peronospora* clade. The *Plasmopara* downy mildew species are in a different clade that is more closely related to the *Phytophthora* species used in this study. The separation between the *Peronospora* lineage and the *Phytophthora*/*Plasmopara* lineages is well supported with a bootstrap value of 0.75. This clustering pattern is in line with the recent studies that suggest that the downy mildew species are not monophyletic within the Peronosporales [2, 71]. The *Phytophthora* species, although belonging to three different *Phytophthora* clades, are more closely related to each other than to the other species in this study. *Phytopythium vexans* appears as a sister group to the *Phytophthora/Peronospora* lineage, which is in line with a recently published multi gene phylogeny [72]. The other five species of *Pythium* form two clusters, as previously observed [72]. The two *Albugo* species form a cluster that is separated from the other clades with maximum bootstrap support.

Based on the core ortholog protein tree, we grouped the species into five phylogenetically-related clades; *Hyaloperonospora/Peronospora*, *Plasmopara*, *Phytophthora*, *Pythium* and *Albugo* for further analysis of the secretomes. Three of these clades only have obligate biotrophic species (*Hyaloperonospora/Peronospora*, *Plasmopara* and *Albugo*), whereas the *Phytophthora* cluster consists of hemi-biotrophs and *Pythium* cluster of necrotrophic species. (*Phyto)Pythium vexans* was included in the *Pythium* cluster. The fish-infecting oomycete *Saprolegnia parasitica* served as an outgroup for the phylogenetic tree and is not used for further comparison.

#### Secretome comparison

For each species, the total number of proteins and the subset that is predicted to be secreted (signal peptide, no additional transmembrane domains, no ER retention signal) is shown in Table 3. *Phytophthora* species generally have a larger proteome than downy mildew species and secrete a larger percentage of the predicted proteins. The *Phytophthora* species in this study are predicted to secrete 1976 proteins on average, whereas the *Plasmopara* and *Peronospora* species secrete an average of 1461 and 703 proteins, respectively.

#### Carbohydrate active enzymes and Pfam domains

The secretome content was compared between species by looking at the carboydrate-active enzymes (CAZymes) and Pfam domains. CAZymes are, amongst others, involved in degrading and modifying plant cell walls, which is an important part of the infection process. The Pfam domain database represents a broad collection of protein families, including RxLR effectors, with diverse functions.

A total of 95 different CAZyme domains were found in the combined secretomes of the 18 oomycete species. The total number of CAZymes per species ranges from 35 in *A*. *laibachii* to 336 in *P*. *sojae*, and was lower in obligate biotrophic species (35–193) compared to *Phytophthora* species (197–336) (S7 Table). A total of 1354 different Pfam domains were found in the combined secretomes of the oomycetes analyzed. The number of domains identified ranged from 304 in *Al*. *candida* to 1710 in *Ph*. *parasitica*. The total number as well as the relative number of Pfam domains in secretomes of obligate biotrophic species was lower in obligate biotrophic species compared to *Phytophthora* and *Pythium* (S8 Table).

**Fig 6:**
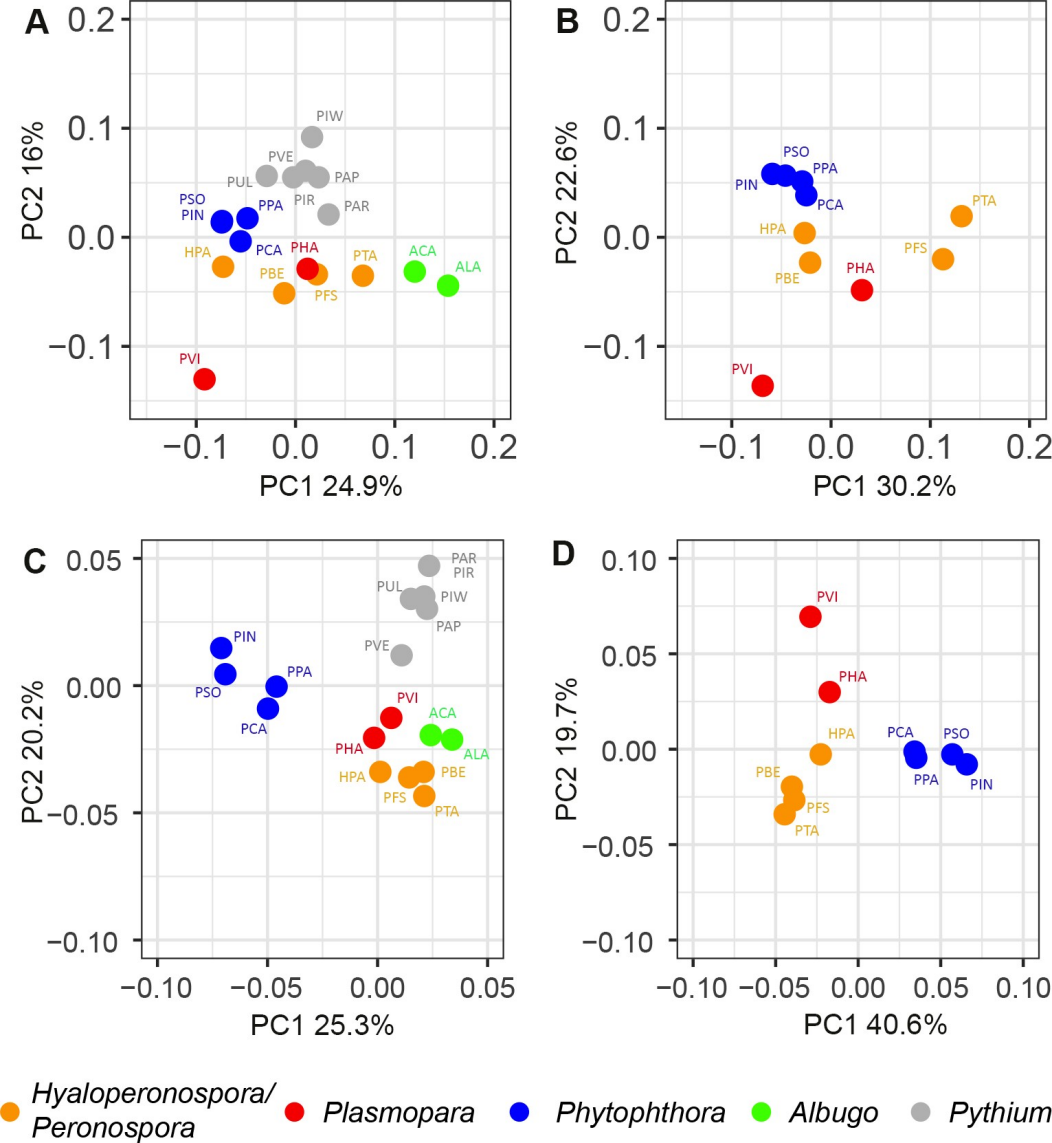
Principal component analysis (PCA) of variation in the relative abundance of secreted CAZymes and Pfam domains. The variation in secreted CAZyme (AB) and Pfam (CD) domains along PC1 and PC2 is depicted in the figure. The PCAs include all of the 18 species (AC) or the *Peronospora*, *Plasmopara* and *Phytophthora* species only (BD). The PERMANOVA test shows that the grouping based on the CAZyme and Pfam domains is significant (P < 0.001). Species are grouped by color based on the classes that were defined in the phylogenetic tree (Fig 5). *Phytophthora* (blue), *Peronospora* (yellow), *Plasmopara* (red), *Albugo* (green) and *Pythium* (grey). Abbr. *PFS; Peronospora (P*.*) effusa*, *PBE; P*. *belbahrii*, *PTA; P*. *tabacina*, *HPA*, *Hyaloperonospora arabidopsidis*, *PHA; Plasmopara (Pl*.*) halstedii*, *PVI; Pl*. *vitiocola*, *PIN; Phytophthora (Ph*.*) infestans*, *PSO; Ph*. *sojae*, *PCA; Ph*. *capsici*, *PPA; Ph*. *parasitica*, *ACA; Albugo (A) candida*, *ALA; A*. *laibachii*, *PUL; Pythium (Py*.*) ultimum*, *PAR; Py*. *arrhenomanes*, *PAP; Py*. *Aphanidermatum*, *PIR; Py*. *irregulare*, *PIW; Py*. *Iawyamai*, *PVE; Phytopythium vexans*.

The presence and numbers of CAZyme and Pfam domains were compared between species using a Principal Component Analysis (PCA), a statistical reduction technique that determines what variables contribute most to the variation observed in a data set. We report the relative abundance of each CAZyme/Pfam domain to the total number of secreted Pfam/CAZyme domains per species, to account for the large variation in absolute numbers of proteins between the species (Fig 6). A PCA based on the absolute numbers can be found in S4 Fig, which shows a similar pattern. The species clusters as depicted in Fig 6 were confirmed using a PERMANOVA (p < 0.001).

The CAZymes-based PCA supports the separate clusters of *Albugo*, *Phytophthora* and *Pythium* species as found in the core ortholog tree (Fig 5). Remarkably, neither the *Hyaloperonospora/Peronospora* nor the *Plasmopara* species form a clear cluster, although the clustering is significant (PERMANOVA p < 0.001). The variation along PC1 (*Hyaloperonospora/Peronospora)* and PC2 (*Plasmopara*) indicates that the secreted CAZyme domains vary largely between the species in these groups, despite their close phylogenetic relationship and same lifestyle. The secreted CAZymes of the two *Plasmopara* species appear more similar to those of the *Hyaloperonospora/Peronospora* species than to the *Phytophthora* species, which is different from the results of the core ortholog protein comparison as shown in the phylogenetic tree (Fig 5). To exclude the effect of the more distantly-related species on the separation between the downy mildew and *Phytophthora* species, the PCA was performed on the set without the *Pythium* and *Albugo* species (Fig 6B). The pattern, as observed in the total set, is maintained when the more distantly related species are excluded from the analysis.

To look further into the properties of the secreted CAZymes we highlighted literature-curated domains of phytopathogenic oomycetes that are known to modify the main plant cell wall components; lignin, cellulose and hemicellulose [69] (S7 Fig). We found that the secretomes differ more in terms of the absolute number of plant cell wall-degrading enzymes than in the relative occurrence of the different corresponding CAZyme catalytic activities per species. Secretomes of obligate biotrophic and hemibiotrophic/necrotrophic oomycetes have secreted proteins with similar functions (like breakdown of cellulose, pectin, hemicellulose etc.) but the numbers and diversity of those proteins in obligate biotrophic species are reduced.

**Fig 7:**
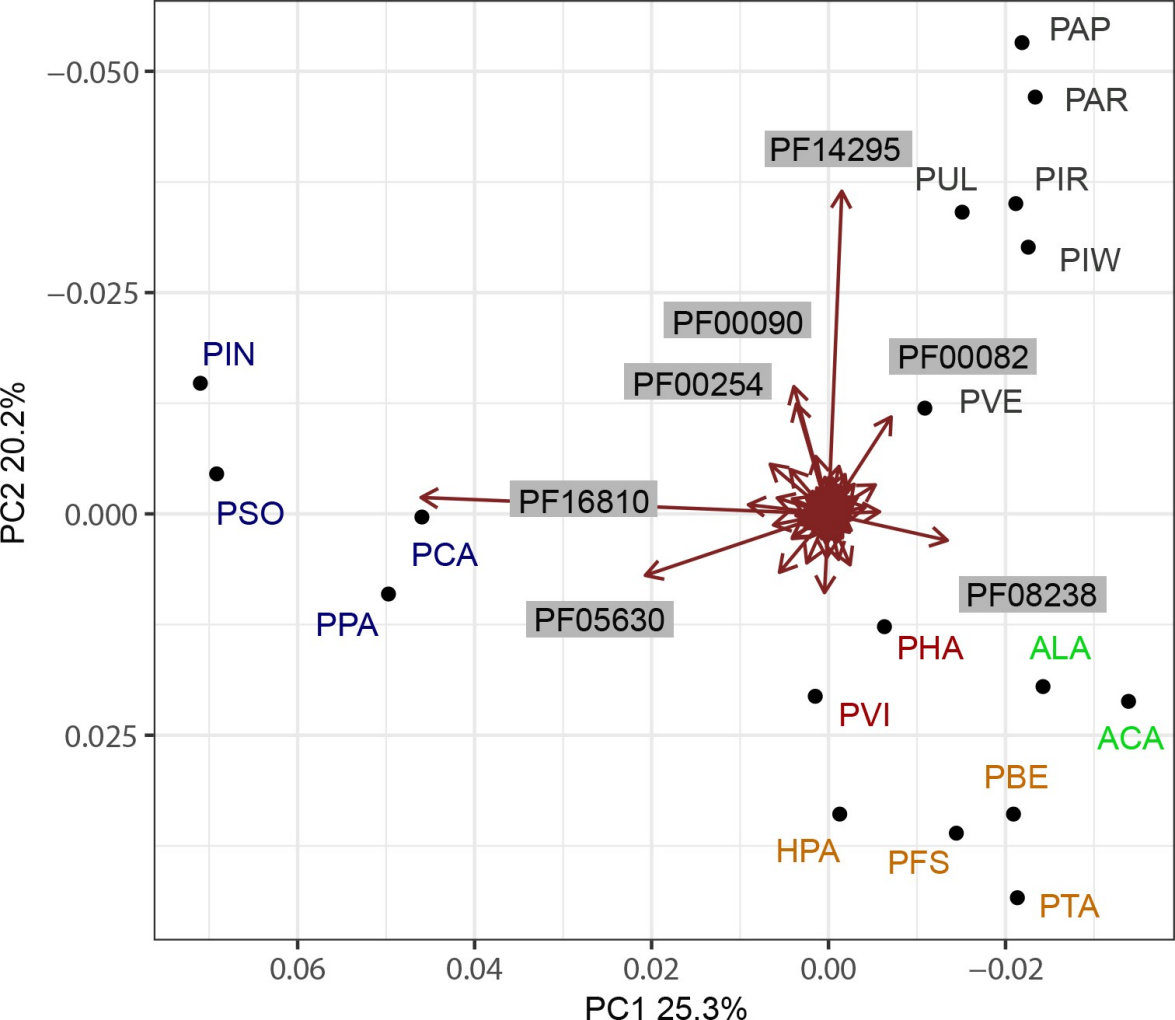
Pfam domains that strongly contribute to the variation in the relative abundance between species. Although many domains contribute to the variation, PF16810, PF05630, PF08238, PF14295, PF00090, PF00254 and PF00082 are the domains that contribute most, as evidenced by the length of their vectors in the biplot.

The Pfam-based PCA shows a clear separation between lifestyles (Fig 6C and 6D). The *Phytophthora* species cluster together and separate from all other species along PC1 (25,3%). The *Pythium* species form a cluster that separates clearly from the other species along PC2 (20,2%). All biotrophic species, including both groups of downy mildews and the *Albugo* species, cluster together. Within the obligate biotrophic cluster the phylogenetic groups (*Hyaloperonospora/Peronospora*, *Plasmopara*, *Albugo)* as found in the core ortholog tree are still present but the differences are minor. To exclude the effect of the more distantly related species on the separation between the obligate biotrophs, the PCA was also performed without *Pythium* and *Albugo* species (Fig 7B). The pattern observed in Fig 6C is maintained when the more distantly related species are excluded from the analysis (Fig 6D).

The repertoires of Pfam domains in the different groups of obligate biotrophs (*Hyaloperonospora/Peronospora*, *Plasmopara* and *Albugo)* are more similar than would be expected based on their taxonomic relationship. This could be the result of convergent evolution towards the obligate biotrophic lifestyle. *Plasmopara* and *Hyaloperonospora/Peronospora* CAZyme repetoires are similar as well, but the *Albugo* species have a different CAZyme profile.

We conclude that a different composition and abundance in secreted Pfam domains is clearly associated with obligate biotrophy, suggesting it is the result of convergent evolution towards an obligate lifestyle.

To look further into the properties of the secreted CAZymes we highlighted literature-curated domains of phytopathogenic oomycetes that are known to modify the main plant cell wall components; lignin, cellulose and hemicellulose [73] (S7 Fig). We found that the secretomes differ more in terms of the absolute number of plant cell wall-degrading enzymes than in the relative occurrence of the different corresponding CAZyme catalytic activities per species. Secretomes of obligate biotrophic and hemibiotrophic/necrotrophic oomycetes have secreted proteins with similar functions (like breakdown of cellulose, pectin, hemicellulose etc.) but the numbers and diversity of those proteins in obligate biotrophic species are reduced.

### Five Pfam domains contribute largely to the difference between obligate biotrophs and others

The Pfam domains that contribute to the variance in PC1 and PC2 were (Fig 6C and 6D) identified using a biplot. In a biplot, the variables are presented as vectors, with their length reflecting their contribution. Many of the domains contribute to the differences between the biological groups, but seven of them stand out (Fig 7. and Table 4).

**Table 4:** Pfam domains that contribute most to the variation between species in the PCA.

	*Hpa/Peronospora*				*Plasmopara*		*Phytophthora*				*Pythium*						*Albugo*	
	Pfs	Pta	Pbe	Hpa	Pha	Pvi	Pin	Ppa	Pso	Pca	Par	Pap	Pir	Piw	Pve	Pul	Aca	Ala
PF16810 RxLR	3	0	0	2	2	0	92	90	104	41	0	0	0	0	0	0	0	0
PF05630 NPP1	7	18	2	10	15	10	31	54	59	42	4	3	3	2	5	7	0	0
PF08238 Sel1 repeat	16	39	14	7	10	23	14	20	22	16	27	30	27	24	10	25	6	13
PF14295 PAN/Apple	1	1	2	0	3	0	39	36	31	22	64	60	35	33	21	33	1	5
PF00082 Subtilase	1	1	1	1	5	20	5	4	2	0	10	21	19	17	9	26	2	2
PF00090 Thrombosp.	0	0	0	0	0	12	14	11	40	12	0	26	21	22	12	23	0	0
PF00254 FKBP	1	13	1	1	1	2	1	1	1	1	2	1	0	2	1	1	0	1

Numbers represent the number of domains per secretome per species. Domains that are relatively less abundant are blue, domains that occur in relatively high numbers are yellow.

Two Pfam domains that have a higher relative abundance in *Phytophthora* contribute strongly to the separation between *Phytophthora* and the other species. The first, PF16810, represents a RxLR protein family with a conserved core α-helical fold (WY-fold). Some of the proteins that this domain was based on have a known avirulence activity [52], i.e. they are recognized by plant resistance proteins. On average, 82 PF16810 domains were identified in *Phytophthora* species compared to 1.3 in *Peronospora*, 1.0 in *Plasmopara* and none in *Albugo* species. Using HMMer searches, many more WY-fold proteins can be identified in *Plasmopara* and *Hyaloperonospora/Peronospora* downy mildew species. However, these proteins do not match to the PF16810 Pfam domain that is based on a larger protein sequence as the HMM.

The second, PF05630, is a necrosis-inducing protein domain (NPP1) that is based on a protein of *Ph*. *parasitica* [74]. This domain is conserved in proteins belonging to the family of Nep1-like proteins (NLPs) that occur in bacteria, fungi and oomycetes [75]. Infiltration of cytotoxic NLPs in eudicot plant species results in cytolysis and cell death, visible as necrosis [76]. *Phytophthora* species are known to have high numbers of recently expanded NLP genes in their genomes, encoding both cytotoxic and non-cytotoxic NLPs [75]. *H*. *arabidopsidis* and other obligate biotrophs tend to have lower numbers and only encode non-cytotoxic NLPs [75, 77].

Domain PF08238 contributes to the distance between the *Phytophthora* and obligate biotrophic species and is relatively more abundant in the biotrophs (PC1). PF08238 is a Sel1 repeat domain that is found in bacterial as well as eukaryotic species. Proteins with Sel1 repeats are suggested to be involved in protein or carbohydrate recognition and ER-associated protein degradation in eukaryotes [78]. No function of proteins with a PF08238 domain is known for oomycete or fungal pathogens.

The distance between *Pythium* and the obligate biotrophic species along PC2 is largely caused by differences in four domains that are commonly reported in oomycete secretomes [71]. The first, PF14295, a PAN/Apple domain, is known to be associated with carbohydrate-binding module (CBM)-containing proteins that recognize and bind saccharide ligands in *Ph*. *parasitica*. Loss of these genes, as in the biotrophs, may facilitate the evasion of host recognition as some CBM proteins are known to induce plant defense [79]. Second, PF00082, is a subtilase domain, which is found in a family of serine proteases. Secreted serine proteases are ubiquitous in secretomes of plant pathogens [80]. Secreted proteases from fungal species have been shown to enhance infection success by degrading plant derived antimicrobial proteins [81]. A third is PF00090, a Thrombospondin type 1 domain that is present in large numbers in the secretome of *Phytophthora* and *Pythium* species but is absent from the secretomes of *Hyaloperonospora/Peronospora* species and *Plasmopara halstedii*. The function of proteins with this domain in oomycetes or plants is unknown. Finally, PF00254 contributes to the separation along PC1, which seems mainly caused by 13 occurences of the domain in the secretome of *P*. *tabacina* versus 2 or less in the secretomes of the other oomycete species.

#### Over and under-representation of Pfam domains in obligate biotrophic species

Statistical analysis of enrichment of Pfam domains, to identify under- and over-represented domains in each group (*Hyaloperonospora/Peronospora*, *Plasmopara*, *Albugo*) compared to *Phytophthora*, confirmed the pattern that was shown in the biplot. In total, 60 Pfam domains were found to be differentially abundant in obligate biotrophic species clusters compared to *Phytophthora* (Table 5). All of the seven Pfam domains that contributed most to the separation between phylogenetic groups in the PCA (Fig 7 and Table 4) were also found to be differentially abundant in at least one obligate biotrophic cluster compared to *Phytophthora* in the enrichment analysis.

**Table 5:** Over and under-representation of Pfam domains in the secretomes of *Hyaloperonospora/Peronospora (HP)*, *Plasmopara (Pl)* and *Albugo (Al)* compared to *Phytophthora* species.

Pfam	Name	Interpro^#^	HP	Pl	Al
PF16810	RxLR protein, Avirulence activity	IPR031825	8,30E-24	2,70E-16	3,90E-07
PF14295	PAN domain	IPR003609	1,80E-07	1,80E-04	16,913
PF00090	Thrombospondin type 1	IPR000884	4,50E-05	77,4987	1,98225
PF05630	Necrosis inducing protein (NPP1)	IPR008701	1,60E-01	1,36788	2,13E-03
PF08238	Sel1 repeat	IPR006597	1,70E-07	4,61559	1,07208
PF00254	FKBP-type cis-trans isomerase	IPR001179	1,80E-04		
PF00050	Kazal-type serine protease inhibitor	IPR002350	2,05E-03		
PF07974	EGF-like domain	IPR013111	1,23E-02		
PF13456	Reverse transcriptase-like	IPR002156	2,60E-10		
PF00300	Histidine phosphatase superfamily	IPR013078	7,74E-03		
PF00665	Integrase core domain	IPR001584	1,07E-02		
PF00571	CBS domain	IPR000644	1,66E-02		
PF00089	Trypsin	IPR001254		1,10E-12	
PF01833	IPT/TIG domain	IPR002909		8,00E-12	
PF00082	Subtilase family	IPR000209		3,10E-10	
PF01341	Glycosyl hydrolases family 6	IPR016288		3,30E-05	
PF00182	Chitinase class I	IPR000726		2,60E-04	
PF01670	Glycosyl hydrolase family 12	IPR002594		1,09E-03	
PF03184	DDE superfamily endonuclease	IPR004875			2,40E-06
PF09818	Predicted ATPase of the ABC class	IPR019195			4,60E-06
PF00169	PH domain	IPR001849			8,10E-06
PF01764	Lipase (class 3)	IPR002921			1,30E-04
PF00026	Eukaryotic aspartyl protease	IPR033121			2,30E-04
PF13405	EF-hand domain	IPR002048			3,70E-04
PF15924	ALG11 mannosyltransferase	IPR031814			3,70E-04
PF01546	Peptidase family M20/M25/M40	IPR002933			3,70E-04
PF07687	Peptidase dimerisation domain	IPR011650			3,70E-04
PF03870	RNA polymerase Rpb8	IPR005570			3,70E-04
PF13041	PPR repeat family	IPR002885			3,70E-04
PF00443	Ubiquitin carboxyl-terminal hydrolase	IPR001394			1,38E-03
PF10152	Subunit CCDC53 of WASH complex	IPR019309			1,63E-03
PF00041	Fibronectin type III domain	IPR003961			2,29E-03
PF07727	Reverse transcriptase	IPR013103			6,09E-03
PF04130	Spc97 / Spc98 family	IPR007259			2,15E-02
PF01753	MYND finger	IPR002893			2,15E-02
PF03577	Peptidase family C69	IPR005322			2,15E-02
PF03388	Legume-like lectin family	IPR005052			3,02E-02
PF03133	Tubulin-tyrosine ligase family	IPR004344			3,02E-02
PF13181	Tetratricopeptide repeat	IPR019734			3,02E-02
PF01156	Nucleoside hydrolase	IPR001910			3,02E-02
PF06367	Diaphanous FH3 Domain	IPR010472			3,02E-02
PF04910	Transcriptional repressor TCF25	IPR006994			3,02E-02
PF00044	Glyceraldehyde 3-ph. dehydrogenase	IPR020828			3,02E-02
PF02800	Glyceraldehyde 3-ph. dehydrogenase	IPR020829			3,02E-02
PF01428	AN1-like Zinc finger	IPR000058			3,02E-02
PF00766	Electron transfer FAD-binding domain	IPR014731			3,02E-02
PF01012	Electron transfer flavoprotein domain	IPR014730			3,02E-02
PF03690	UPF0160 (uncharacterized)	IPR003226			3,02E-02
PF13307	Helicase C-terminal domain	IPR006555			3,02E-02
PF08683	Microtubule-binding calmodulin-reg	IPR014797			3,02E-02
PF01846	FF domain	IPR002713			3,02E-02
PF13418	Galactose oxidase				3,02E-02
PF03776	MinE	IPR005527			3,02E-02
PF13815	Iguana/Dzip1-like DAZ-interacting	IPR032714			3,02E-02
PF04851	Type III restriction enzyme	IPR006935			3,02E-02
PF13831	PHD-finger				3,02E-02
PF04045	Arp2/3 complex, p34-Arc	IPR007188			3,02E-02
PF08144	CPL (NUC119) domain	IPR012959			3,02E-02
PF00659	POLO box duplicated region	IPR000959			3,02E-02
PF08450	SMP-30/Gluconolaconase/LRE-like	IPR013658			4,76E-02

Over (green) and under (blue)-representation was tested relative to the expected distribution of each Pfam domain. The abundance of each domain was compared between the species clusters using a Chi-square test with Bonferroni correction. Bonferroni corrected p-values are shown in the table.

^#^The InterPro domain code corresponding to each Pfam domain is provided.

Previous studies identified Pfam domains that are associated with virulence in other phytopathogenic oomycete species like *Pythium*, *Plasmopara*, *Peronospora* and *Phytophthora* [82]. The occurrence of these known virulence-associated domains in the *Pfs* proteome is summarized in S5 Fig. We found that obligate biotrophic species have a lower total, as well as relative, number of secreted proteins with virulence-associated domains compared to the other oomycete species.

#### Host-translocated effectors

**Fig 8:**
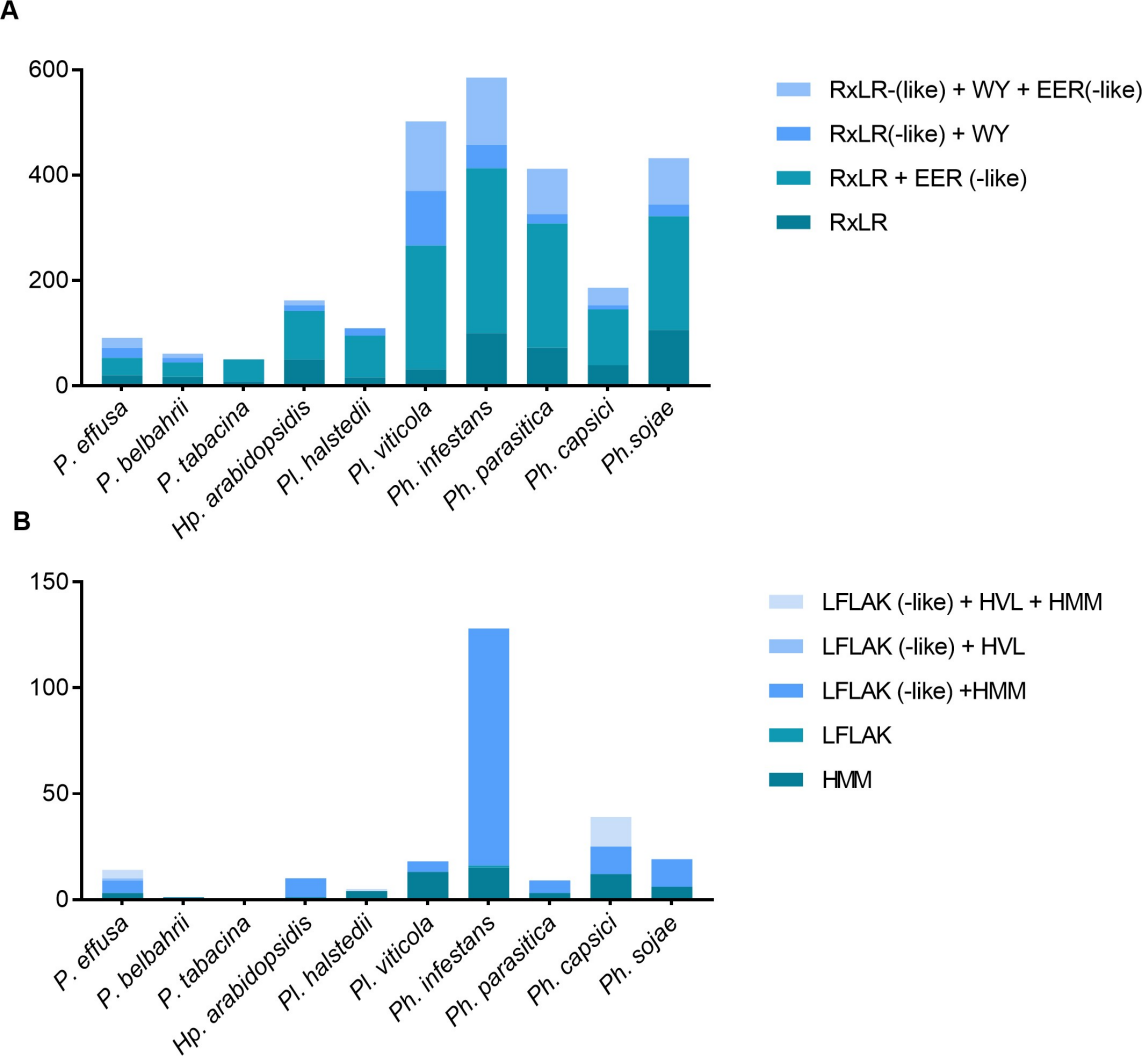
Predicted (a) RxLR and (b) CRN effectors in the secretome of *Hyaloperonospora/Peronospora*, *Plasmopara* and *Phytophthora* species. The predicted effectors are classified into four (RxLR) or five (CRN) categories, based on the additional domains they possess. Please note that the number of *Pfs* effectors is slightly different from the numbers reported before (S2 and S3 Figs). For this comparison we used HMM models that were previously published rather than the models trained for *Pfs* (S2 and S3 Figs).

The RxLR effector models in the Pfam database (PF16810 and PF16829) mentioned above cover only a small fraction of the predicted RxLR effectors in secretomes of phytopathogenic oomycetes. We predicted the total number of host-translocated effectors for each secretome using a Perl regex script and HMM searches (see methods), including RxLR effectors without WY domains and CRN effectors (Fig 8). RxLR effector proteins were more abundant in *Phytophthora* compared to the obligate biotrophic species. On average 399 RxLR effector proteins were found in *Phytophthora* whereas *Plasmopara* and *Hyaloperonospora/Peronospora* had 79 and 90. The same pattern is evident for CRN effectors. The average number of CRN proteins in *Hyaloperonospora/Peronospora* is 11, while *Plasmopara* has 12 and *Phytopthora* 56. We conclude that downy mildew species (*Hyaloperonospora/Peronospora* and *Plasmopara*) have fewer host-translocated effectors compared to *Phytophthora* species.

## Discussion

### Taxonomic filtering

The ability to sequence full genomes at high pace and relatively low cost has aided research in phytopathology dramatically. Over the past few years, the genomes of many phytopathogenic oomycetes have been sequenced and their genomes revealed an arsenal of protein coding genes with a putative virulence role. However, technical difficulties restricted the sequencing and assembly of genomes of obligate biotrophic oomycetes that cannot be cultured axenically. Obligate biotrophic species can only grow on living host tissue so when collecting spores for DNA isolation DNA of other microbes and the host plant will inevitably contaminate the sample, which complicates the genome assembly. In this paper we use a metagenome filtering method resulting in the assembly of a relatively clean genome sequence of the obligate biotrophic downy mildew of spinach, *Peronospora effusa*.

To get a clean assembly, sequence that are derived from different species were filtered out and removed. Several methods were considered to identify and filter contigs or reads that were likely contaminants in our data. Initially we considered to filter contigs or reads based on their GC content, since this differs between genomes of oomycetes [83] and many other microbes [84]. However, some bacterial species have a GC content similar to that of Pfs, e.g. *E*. *coli* with a GC content of 51.7% [84]. In addition, the GC content is not constant over the genome, so filtering based on this could potentially remove valuable parts of the genome.

Alternatively, reads of non-oomycete origin could be identified by mapping them to databases with sequences of known taxonomy. For example, a database containing only oomycete or bacterial genomes. This is not ideal as the databases are incomplete and are likely to contain annotation errors. In addition, it could lead to the removal of novel parts of the downy mildew genome that are not present in other oomycetes, and which would hamper the study of valuable species-specific parts of your genome.

The filtering we applied with the CAT tool does not classify a contig based on a single hit. Instead it determines the taxonomic origin of each ORF on an assembled contig or corrected PacBio read, providing a robust classification [15]. In our *Pfs* study, after filtering with the CAT-pipeline of the error-corrected PacBio reads, 50% remained, and were used in the assembly. Of the sequenced (unfiltered) Illumina reads, 56% could be aligned to the final assembly. This indicates that roughly half of our sequencing reads originated from other sources besides *Pfs*. Notably, while the classifications in the original CAT paper were only benchmarked on prokaryotic sequences [15], our study shows that the tool also performs well for classifying eukaryotic contigs. Thus, CAT may also be promising for classification of eukaryotes including oomycetes in metagenomic datasets, provided that long contigs, or corrected PacBio or Nanopore sequencing reads are available.

It should be noted that sequences of unknown taxonomy were maintained for the assembly, making it possible that these are still contaminants. When we compare the taxonomic distributions generated by Kaiju of the pre-assembly and final assembly, we see a dramatic reduction of sequences of bacterial origin (Fig 3). The oomycete content according to Kaiju and the overall GC content of the final assembly is similar to that of genome assemblies of axenically-grown oomycetes. We can therefore conclude that the CAT filtering method, allowed the successful removal of sequences of non-oomycete origin.

### Hybrid assembly

Most oomycete genomes sequenced to date were found to contain long repeat regions [85] that cannot be resolved using only a short-read technology such as Illumina. Long reads can potentially sequence over long repeats, and contribute to the contiguity of a genome assembly [86]. Therefore, our Illumina data was complemented with long read PacBio sequences in an attempt to close gaps between contigs. Although the inclusion of PacBio reads in our assembly improved the contiguity, the final result still consists of a large number of contigs, indicating that our PacBio reads were unable to span many repeat regions. Besides biological reasons for the large number of contigs, there could also be a technical reason. Prior to PacBio sequencing whole genome amplification (WGA) with random primers was performed as the initial sequencing attempt with non-amplified DNA barely yielded sequencing reads. WGA might create a bias, where some parts of the oomycete genome may be under-represented in the PacBio data.

### The genome of *Pfs1*

The assembled *Pfs1* genome size is 32.4 Mb divided over of 8,635 contigs. The genome is highly gene dense and contains in total 13,227 genes. Overall, the BUSCO analysis showed that this assembly contains most of the gene-space. Many of the 8,635 contigs were smaller than 1 kb. However, the CAT filtering method performs best on relatively large contigs containing multiple ORFs. Therefore, small contigs could still contain sequences derived from other organisms. The removal of these small contigs results in only a small genome size reduction (1.9 Mb) and loss of gene models (597), but significantly reduces the number of contigs (by 5,027). When we also account for genes that have a significant overlap (>20%) with repeats in the genome (3983 gene models), or that were annotated as transposable elements (36 gene models that did not had an overlap with a repeat region) we come to 8,976 high-confidence gene models.

The genomes of *Pfs* race *13* and *14* have recently been published [87, 88], with a similar genome size (32.1 Mb, and 30.8 Mb respectively) and gene content (~ 8000 gene models) compared to our *Pfs1* genome assembly. Contrary to our assembly method, the input data for those genome assemblies were filtered by alignment to an oomycete and bacterial database to discard reads that do not belong to the oomycete genus. This filtering method could potentially lead to the incorporation of bacterial sequences that are not in the public databases. Besides, the positive filtering for oomycete scaffolds against NCBI nt database could have resulted in the loss of *Pfs* specific genome sequences. In addition, by filtering reads based on a database containting bacterial and fungal sequences, part of the *Pfs* genome yielded by horizontal gene transfer (HGT) may be discarded [89]. The CAT-tool overcomes this issue by determining the overall taxonomy of larger contigs based on multiple genes.

### *Peronospora* species have reduced genomes

Recent sequencing of *Peronospora* species shows that they have remarkably small and compact genomes (32.3–63.1 Mb) compared to *Phytophthora* (82–240 Mb) species [32, 35, 87, 90]. The *k-*mer analysis predicts the *Pfs1* genome to be 36.2 Mb containing 8.8 Mb of repeats (24%). The predicted genome size of *Pfs R13* and *R14* based on *k-*mer analysis is 44.1–41.2 mb (repeats; 24–22%) [87]. The increased genome size of *Phytophthora* is attributed to an ancestral whole genome duplication in the lineage leading to *Phytophthora* and to an increase in the proportion of repetitive non-coding DNA [32, 91]. The duplication event has been proposed to have taken place after the speciation of *H*. *arabidopsidis* [92]. However new multigene phylogenies show that the *Peronospora* lineage has speciated after the divergence of *Phytophthora* clade 7 from clade 1 and 2. Notably, these three clades all contain species with duplicated genomes [2, 5, 6, 93]. This would suggest that an ancestral whole genome duplication before this speciation point would also apply to *Peronospora*, and would mean that duplication cannot account for the difference in genome size. The availability now of genomes of three *Peronospora* species for comparisons asks for a reevaluation of the timing of the duplication and subsequent speciation events.

Biologically, the question of how *Peronospora* species can be host-specific and obligate biotrophic while maintaining only a small and compact genome is interesting. It is argued that the trend in filamentous phytopathogens is towards large genomes with repetitive stretches to enhance genome plasticity [91]. Plasticity may enable host jumps and adaptations that favor the species for survival over species with small, less flexible genomes [91]. The reduced genomes of *Peronospora* species show an opposing trend that cannot be attributed to their obligate biotrophic lifestyle alone, as it is not evident in *Plasmopara* species (75 Mb– 9 2 Mb) [5, 94]. Sequencing of multiple isolates of the same *Peronospora* species may shed light on genome plasticity at the species level.

### Secretome reflects biotrophic lifestyle

#### Evolving biotrophy

The biotrophic lifestyle has emerged on several independent occasions in the evolution of filamentous plant pathogens, in several branches of the tree of life. Convergent evolution is thought to be the main driving factor behind the development of biotrophy in such distantly related organisms [95]. However, it was shown that horizontal gene transfer can also occur between fungi and oomycetes, resulting in 21 fungal proteins in the secretome of *H*. *arabidopsidis*. Out of these 21 proteins, 13 were predicted to secreted, indicating that horizontal gene transfer may affect a species pathogenicity and interaction with the host [96, 97].

It was proposed that the critical step for adopting biotrophy in filamentous phytopathogens is the ability to create and maintain functional haustoria [98]. To do so, a species needs to be able to avoid host recognition or suppress the host defense response. A proposed mechanism for avoidance of host recognition is the loss of proteins involved in cell wall degradation, as evidenced by the reduction of cell wall degrading enzymes in mutualistic species compared to biotrophs [99]. In this and other studies, we find a reduction of the number of cell wall degrading enzymes in obligate biotrophic species compared to hemi-biotrophic *Phytophthora* species (S5 Fig) [30]. This is true for all three obligate biotrophic groups in this study (*Hyaloperonospora/Peronospora*, *Plasmopara* and *Albugo*) although the difference is less clear in *Plasmopara*. Possibly this reduction is the result of a similar selection pressure to reduce recognition by the host plant in the biotrophic species, where the hemi-biotrophic nature of the interaction between host and Phytophthora allows for slightly less caution in recognition avoidance.

The other mechanism of establishing a strong interaction is suppression or avoidance of the host defense response. Biotrophic infections are often accompanied by co-infection of species that are unable to infect the plant in the absence of the biotroph, indicating efficient defense suppression [98, 100]. We found enhanced numbers of secreted serine proteases (PF00082) (suppression) and reduced numbers of proteins with PAN/Apple domains that are known to be recognized by the plant immune system.

While the expansion of host translocated RxLR effectors is evident in both hemi-biotrophic and biotrophic species, their numbers are smaller in secretomes of obligate biotrophs. CRN effectors are especially reduced in secretomes of biotrophic species. As opposed to RxLR effectors, CRNs are an ancient class of effectors that are known to induce cell death. Obligate biotrophic species presumably lost them as they are not beneficial for their survival.

In this study we first showed that the CAT tool performs well for taxonomic filtering of eukaryotic contigs. We provided a clean reference genome of a race 1 isolate of the spinach infecting downy mildew, *Pfs1*. In a comparative approach, we found that the secretomes of the obligate biotrophic oomycetes are more similar to each other than to more closely related hemi-biotrophic species when comparing the presence and absence of functional domains, including the host translocated effectors. We conclude that adaptation to biotrophy is reflected in the secretome of oomycete species.

## Supporting information

S1 Fig GC plot of various oomycete assemblies on contigs larger than 1kb. (TIF)

S2 Fig RxLR (-like) motifs observed in the putative RxLR effectors identified in the genome of *Pfs1*. For each (degenerate) RxLR motif the presence of a WY domain (orange), EER-like (green) domain or both (purple) is shown.
(TIF)

S3 Fig CRN (-like) motifs observed in the putative CRN effectors identified in the genome of *Pfs1*. For each (degenerate) CRN protein the presence of an HVL domain (orange), identified with an CRN HMM model (red) or both (green).
(TIF)

S4 Fig PCA on absolute numbers of secreted CAZyme domains. (TIF)

S5 Fig Secreted cell wall degrading proteins (CAZymes). Numbers (a) of literature curated plant cell wall degrading enzymes per species. (b) The same data represented as fraction of the total number cell wall degrading protein domains per species.
(TIF)

S6 Fig PCA on absolute numbers of secreted Pfam domains. (TIF)

S7 Fig Secreted pathogenicity associated Pfam domains. Occurrence of Pfam domains known to be involved in pathogenicity within the secretome of each species. Figure (a) shows the absolute number of Pfam domains, while (b) shows the number relative to the total number of Pfam domains per species.
(TIF)

S1 Table Species used for comparative secretomics. (XLSX)

S2 Table Comparison of conserved eukaryotic genes for different oomycetes and the *Pfs1* assembly using BUSCO. (XLSX)

S3 Table Repeat elements in the *Pfs1* genome. Repeat elements identified in the *Pfs1* genome, for each repeat type the total numbers and percentage are shown. In addition, also a detailed annotation for each repeat element is provided.
(XLSX)

S4 Table Genome sizes and repeat content of different assembled oomycete genomes. (XLSX)

S5 Table Putative annotations of the *Pfs* proteins as obtained with ANNIE. In addition, the presence of a N-terminal signal peptide for secretion, WY motif, TM motif and overlap with a repeat region are listed for each protein coding gene.
(XLSX)

S6 Table Overview of the host translocated effectors (RxLR and CRN) identified in the genome of *Pfs1*. Also, their respective functional domains and locations are listed per effector. Selected effectors that were used in the gene intergenic distance analysis are listed in the second tab.
(XLSX)

S7 Table Secreted CAZyme domains per species. (XLSX)

S8 Table Secreted Pfam domains per species. (XLSX)
