# Supplementary figures and images for: Genome reconstruction of the non-culturable spinach downy mildew Peronospora effusa by metagenome filtering
Source: PLoS One. 2020 May 12;15(5):e0225808. doi: 10.1371/journal.pone.0225808 (PMC7217449; doi:10.1371/journal.pone.0225808)

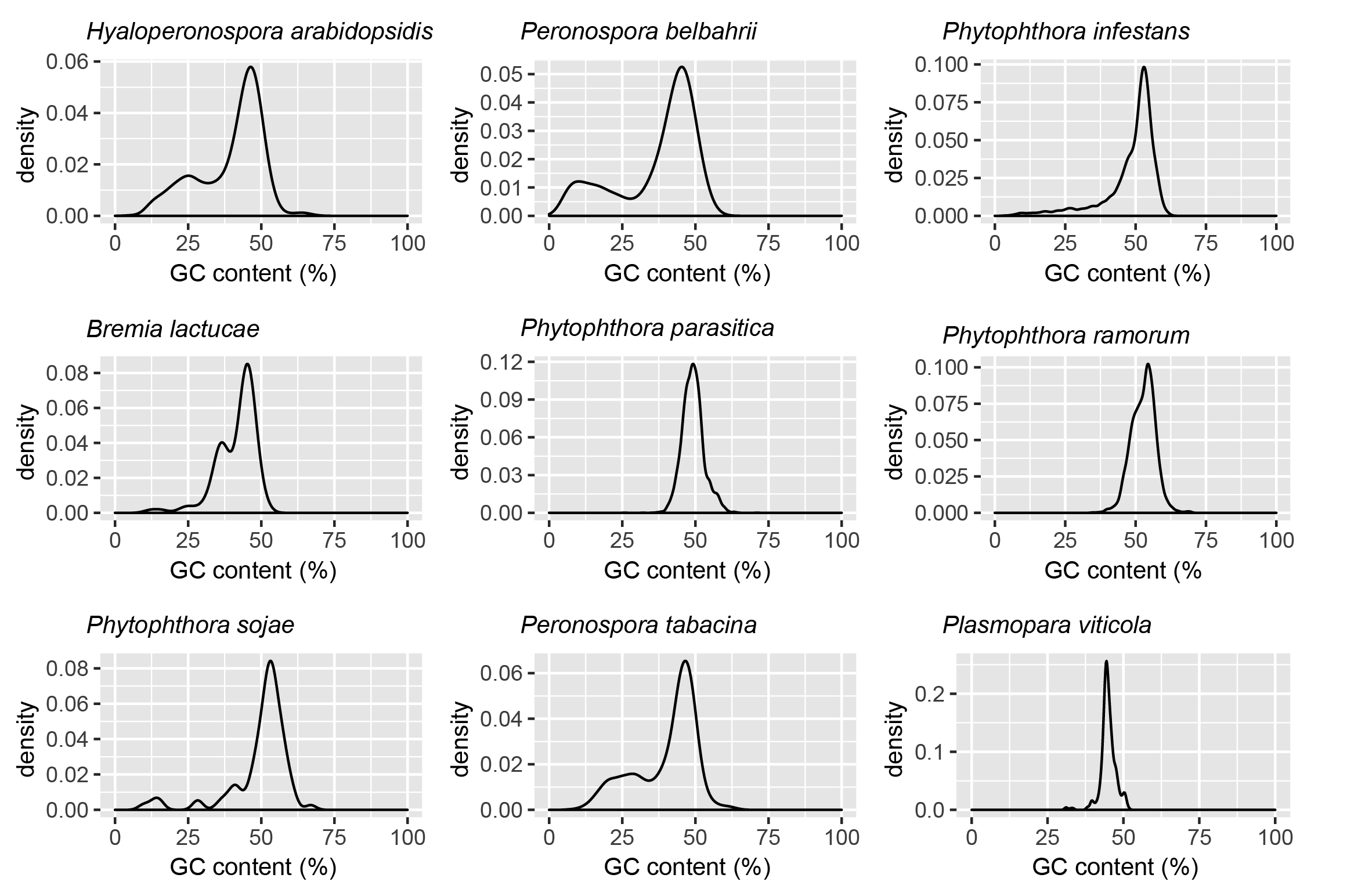

Supplement: S1 Fig — (TIF) [file pone.0225808.s001.tif]

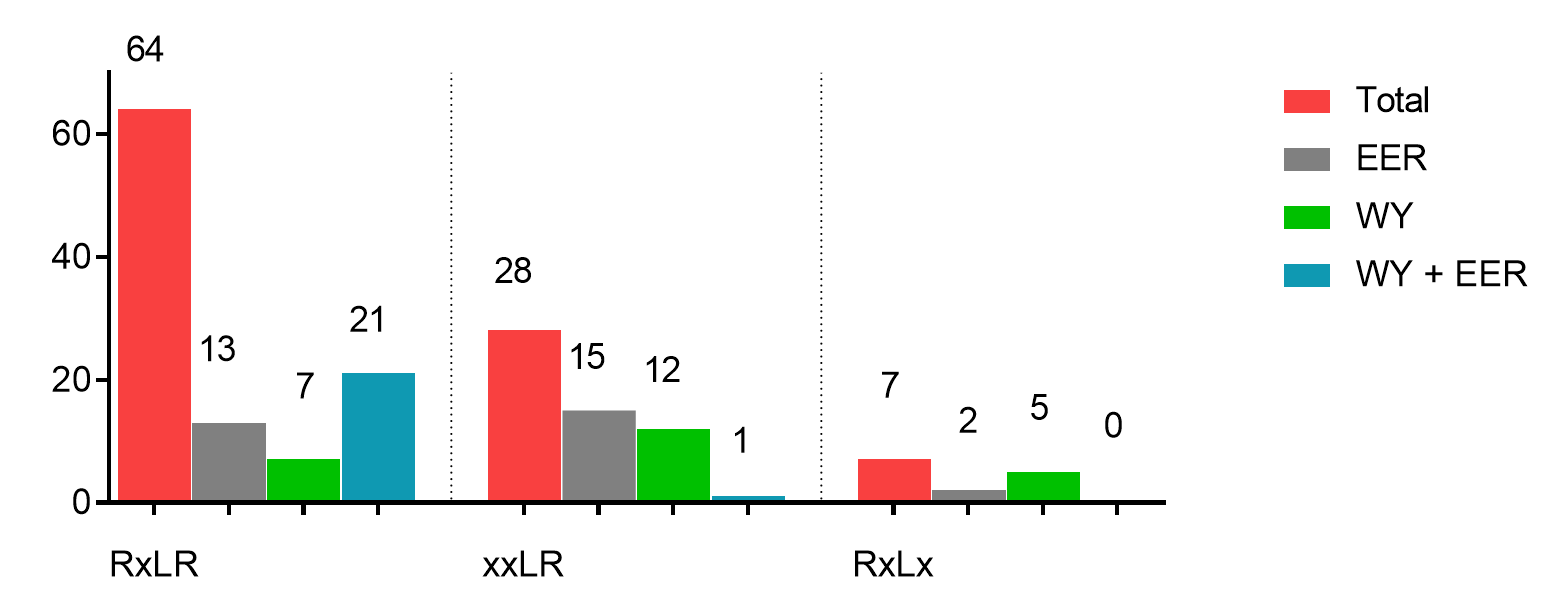

Supplement: S2 Fig — For each (degenerate) RxLR motif the presence of a WY domain (orange), EER-like (green) domain or both (purple) is shown. (TIF) [file pone.0225808.s002.tif]

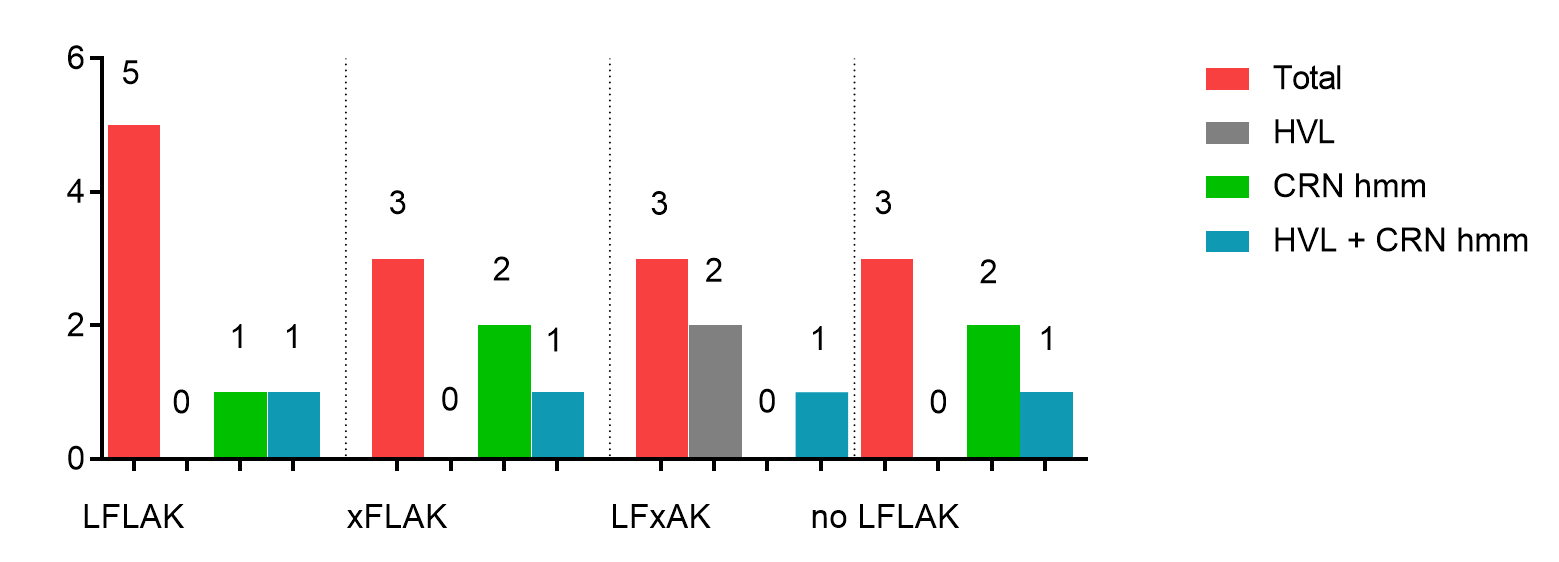

Supplement: S3 Fig — For each (degenerate) CRN protein the presence of an HVL domain (orange), identified with an CRN HMM model (red) or both (green). (TIF) [file pone.0225808.s003.tif]

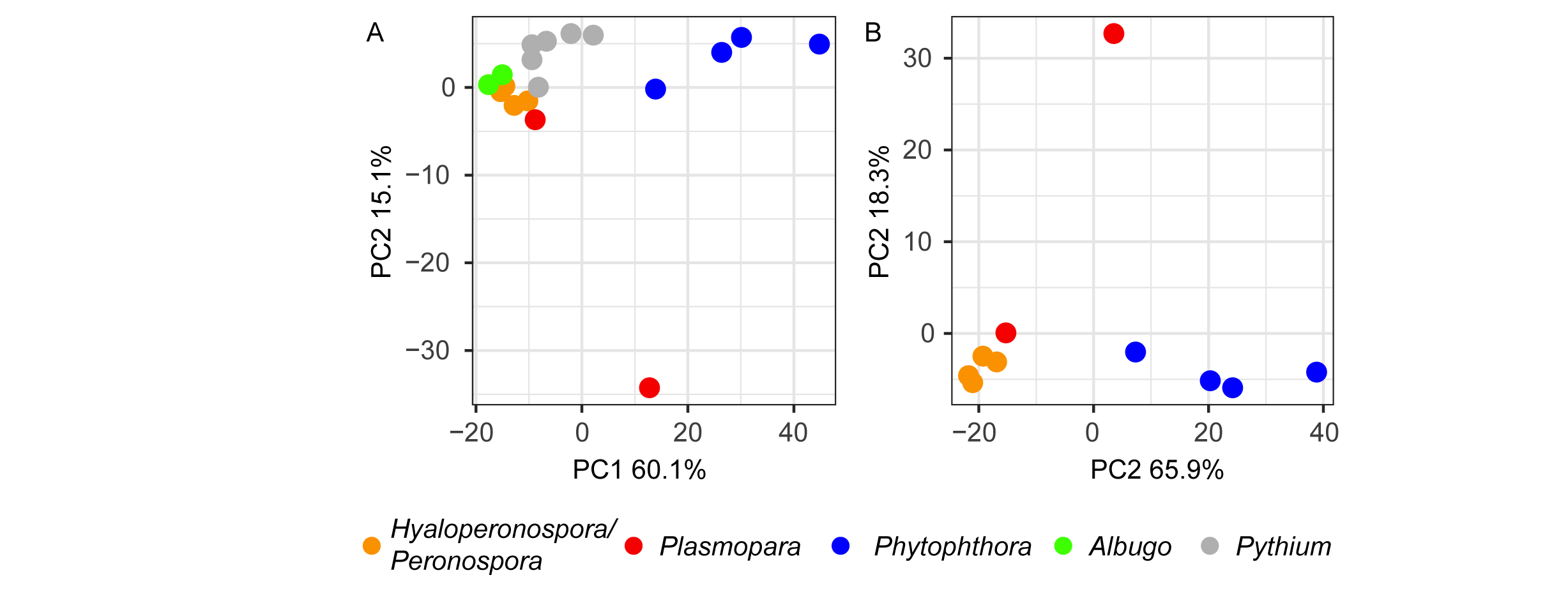

Supplement: S4 Fig — (TIF) [file pone.0225808.s004.tif]

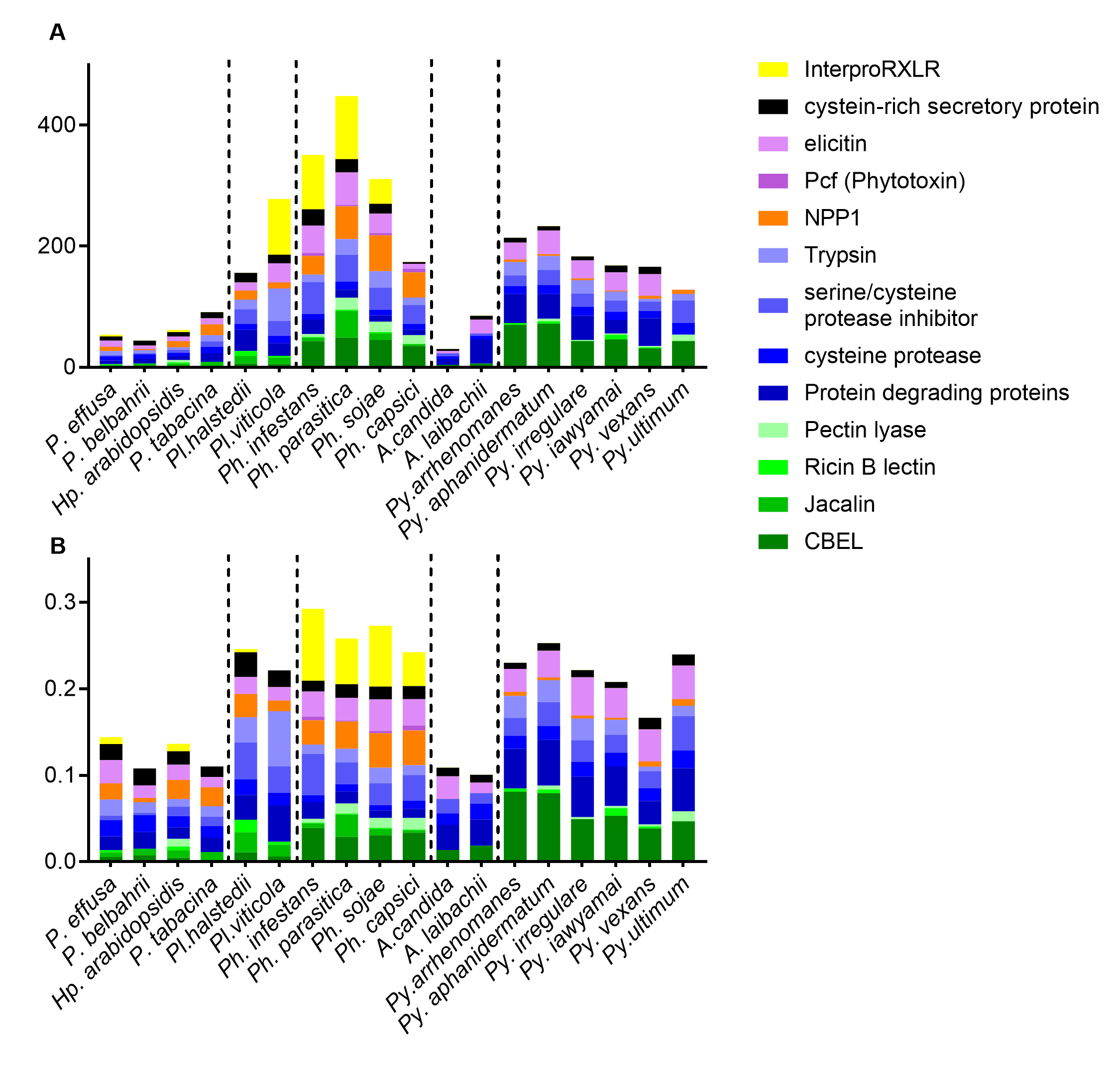

Supplement: S5 Fig — Numbers (a) of literature curated plant cell wall degrading enzymes per species. (b) The same data represented as fraction of the total number cell wall degrading protein domains per species. (TIF) [file pone.0225808.s005.tif]

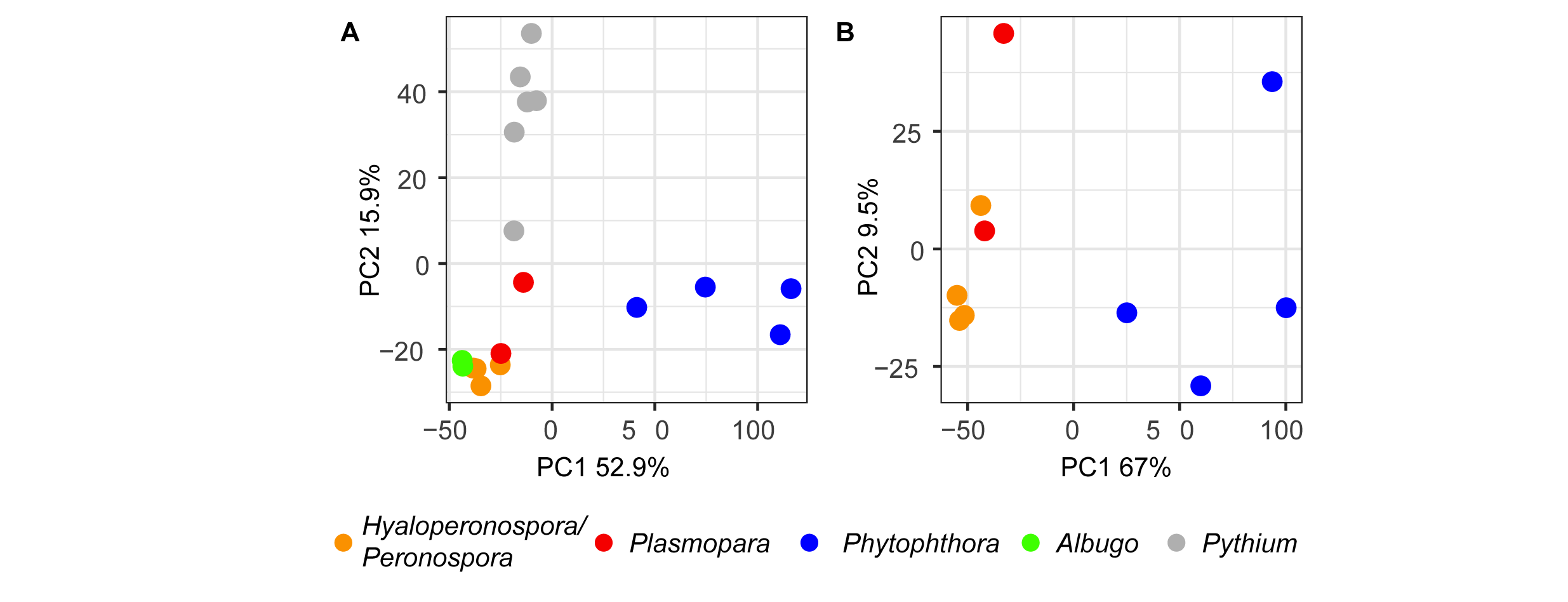

Supplement: S6 Fig — (TIF) [file pone.0225808.s006.tif]

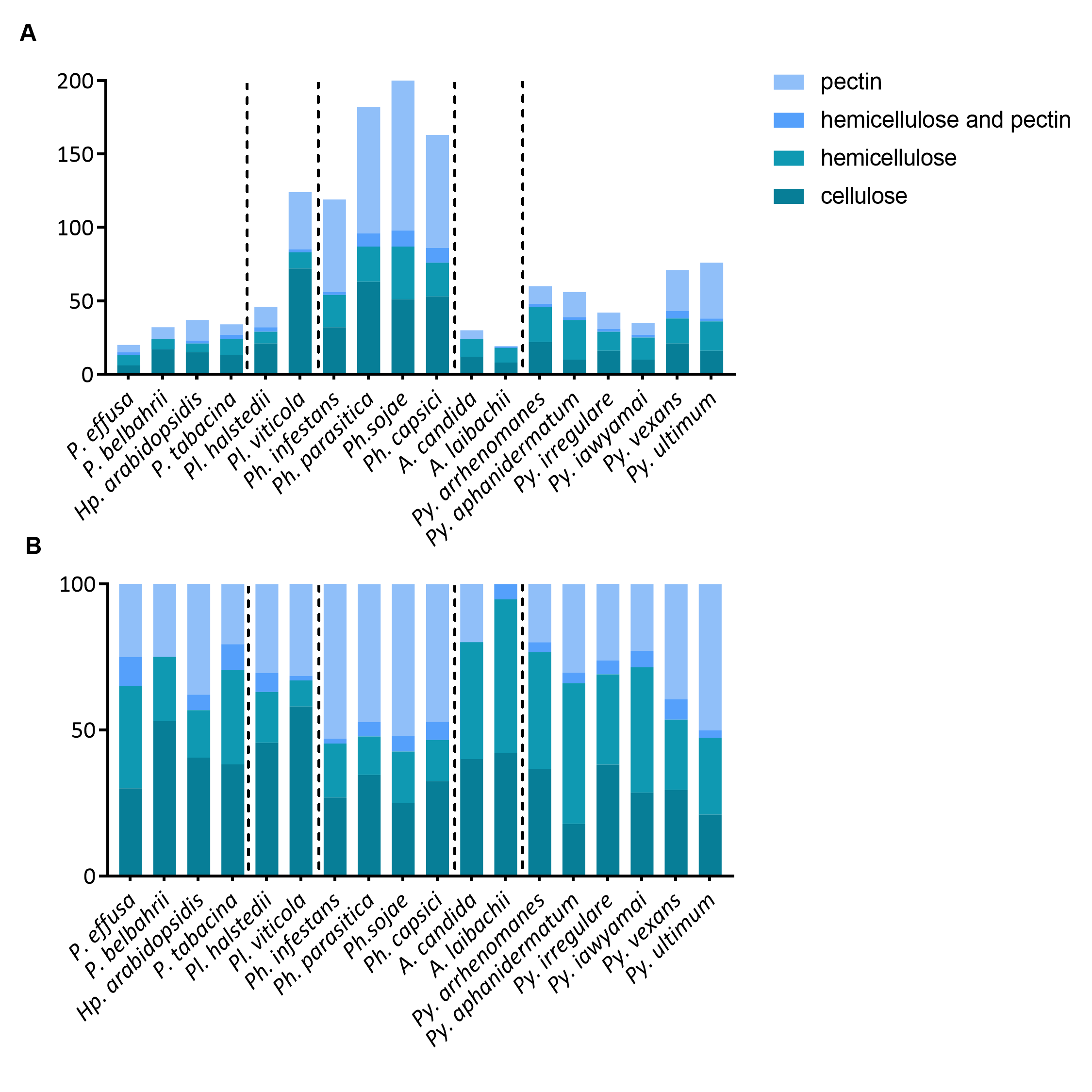

Supplement: S7 Fig — Occurrence of Pfam domains known to be involved in pathogenicity within the secretome of each species. Figure (a) shows the absolute number of Pfam domains, while (b) shows the number relative to the total number of Pfam domains per species. (TIF) [file pone.0225808.s007.tif]
